# Chromosome-level genome assemblies and genetic maps reveal heterochiasmy and macrosynteny in endangered Atlantic *Acropora*

**DOI:** 10.1186/s12864-024-11025-3

**Published:** 2024-11-20

**Authors:** Nicolas S. Locatelli, Sheila A. Kitchen, Kathryn H. Stankiewicz, C. Cornelia Osborne, Zoe Dellaert, Holland Elder, Bishoy Kamel, Hanna R. Koch, Nicole D. Fogarty, Iliana B. Baums

**Affiliations:** 1 Department of Biology, The Pennsylvania State University, University Park, PA USA; 2 Department of Marine Biology, Texas A&M University at Galveston, Galveston, TX USA; 3 Institute for Systems Biology, Seattle, WA USA; 4 Australian Institute of Marine Science, Townsville, QLD Australia; 5 Lawrence Berkeley National Laboratory, Joint Genome Institute, Berkeley, CA USA; 6 Mote Marine Laboratory, Coral Reef Restoration Program, Summerland Key, FL USA; 7 Department of Biology and Marine Biology, University of North Carolina Wilmington, Wilmington, NC USA; 8 Helmholtz Institute for Functional Marine Biodiversity at the University of Oldenburg (HIFMB), Heerstraße 231, Oldenburg, Ammerländer 26129 Germany; 9 Alfred Wegener Institute, Helmholtz-Centre for Polar and Marine Research (AWI), Am Handelshafen, Bremerhaven, Germany; 10 Institute for Chemistry and Biology of the Marine Environment (ICBM), School of Mathematics and Science, Carl Von Ossietzky Universität Oldenburg, Ammerländer Heerstraße 114-118, Oldenburg, 26129 Germany

**Keywords:** Acropora, Coral, Genome, Chromosome, Ancestral linkage group, Linkage map, Recombination rate, Heterochiasmy, Hermaphrodite

## Abstract

**Background:**

Over their evolutionary history, corals have adapted to sea level rise and increasing ocean temperatures, however, it is unclear how quickly they may respond to rapid change. Genome structure and genetic diversity contained within may highlight their adaptive potential.

**Results:**

We present chromosome-scale genome assemblies and linkage maps of the critically endangered Atlantic acroporids, *Acropora palmata* and *A. cervicornis*. Both assemblies and linkage maps were resolved into 14 chromosomes with their gene content and colinearity. Repeats and chromosome arrangements were largely preserved between the species. The family Acroporidae and the genus *Acropora* exhibited many phylogenetically significant gene family expansions. Macrosynteny decreased with phylogenetic distance. Nevertheless, scleractinians shared six of the 21 cnidarian ancestral linkage groups as well as numerous fission and fusion events compared to other distantly related cnidarians. Genetic linkage maps were constructed from one *A. palmata* family and 16 *A. cervicornis* families using a genotyping array. The consensus maps span 1,013.42 cM and 927.36 cM for *A. palmata* and *A. cervicornis*, respectively. Both species exhibited high genome-wide recombination rates (3.04 to 3.53 cM/Mb) and pronounced sex-based differences, known as heterochiasmy, with 2 to 2.5X higher recombination rates estimated in the female maps.

**Conclusions:**

Together, the chromosome-scale assemblies and genetic maps we present here are the first detailed look at the genomic landscapes of the critically endangered Atlantic acroporids. These data sets revealed that adaptive capacity of Atlantic acroporids is not limited by their recombination rates. The sister species maintain macrosynteny with few genes with high sequence divergence that may act as reproductive barriers between them. In the Atlantic *Acropora*, hybridization between the two sister species yields an F1 hybrid with limited fertility despite the high levels of macrosynteny and gene colinearity of their genomes. Together, these resources now enable genome-wide association studies and discovery of quantitative trait loci, two tools that can aid in the conservation of these species.

**Supplementary Information:**

The online version contains supplementary material available at 10.1186/s12864-024-11025-3.

## Background

Corals are early branching metazoans with a long evolutionary history, first appearing in the fossil record 240 Mya, though phylogenomic analyses suggest the earliest scleractinians emerged around 425 Mya [1]. Several genome assemblies are now complete and reveal substantial similarities between early and late branching metazoans [2], with a particularly slow rate of genome rearrangement [3, 4] and mitochondrial mutation rate [5, 6] in the phylum Cnidaria (corals, hydrozoans, and jellyfish) when compared with other metazoans such as bilaterians. Over evolutionary time scales, corals have adapted to changing environments [7], but it is less clear how fast they may adapt to rapid changes. Aspects of adaptive capacity may include the structure of an organism’s genome, the genetic diversity contained within it, and the rate at which genetic diversity is recombined [8].

Corals have complex lifestyles: planktonic larvae settle and form sessile adult colonies via polyp budding and branch fragmentation [9–11]. During annual broadcast spawning events, adult colonies of the two Atlantic *Acropora* species, *A. palmata* and *A. cervicornis*, release egg/sperm bundles into the water column where they dissociate [12]. Self-fertilization is genet-specific and self-fertilizing genets occur at low frequency in the populations of both *A. palmata* and *A. cervicornis* [13–15]. Larvae develop for a few days in the water column before swimming towards the benthos where they settle and metamorphose [16]. Once a primary polyp has formed, symbiotic algae in the order Symbiodiniaceae colonize the coral tissue. Adult colonies of Atlantic acroporids most often harbor the species *Symbiodinium ‘fitti’* [17]*.* Recruitment of sexually produced offspring into adult populations of these acroporids is now rare [18]. Populations of Atlantic acroporids have declined more than 80% in recent decades throughout the Atlantic and Caribbean due to anthropogenic impacts, infectious diseases, and temperature induced bleaching events [19, 20] leading to their current status as a federally listed threatened species under the US Endangered Species Act.

Genome assemblies are now available from all classes of cnidarians [21]. In Anthozoa, the Hexacorallia are represented by dozens of genomes from genera such as *Acropora* [22–24]*, Astrangia* [25], *Exaiptasia* [26]*, Nematostella* [3] and the Octocorallia by at least eight genomes from taxa such as *Renilla* [27]***,**** Dendronephthya* [28]*, Xenia* [29]*,* and *Heliopora* [30]. Seven chromosome-resolved assemblies are published for scleractinian corals [22, 24, 31–33]. While most coral species are diploid, other ploidies exist (e.g. *Pocillopora acuta* [34]*,*). The ancestral cnidarian chromosome number is seventeen [4], whereas coral genomes generally have fourteen chromosomes (2*n* = 28; [35]) and genome sizes are between 300 Mb – 1 Gb (eg., [22, 36–38]). The number of genes is typically 30,000–40,000 with some exceptions (e.g. *Montipora capitata* and *Porites compressa* in [34]).

Genetic diversity fuels adaptation by providing targets for selection (e.g. [39, 40]). Population genetic data indicate that corals are highly heterozygous and contain substantial genetic diversity over their large geographic ranges [41, 42], including the two Atlantic acroporids [43–49]. Hybridization and introgression among coral populations and species is facilitated by external fertilization of embryos and synchronized mass spawning events [7, 50, 51]. Indeed, the two Atlantic acroporids hybridize to form an F1 hybrid and backcrosses of the F1 hybrid into both parent species are observed at a low frequency [47].

**Fig. 1:**
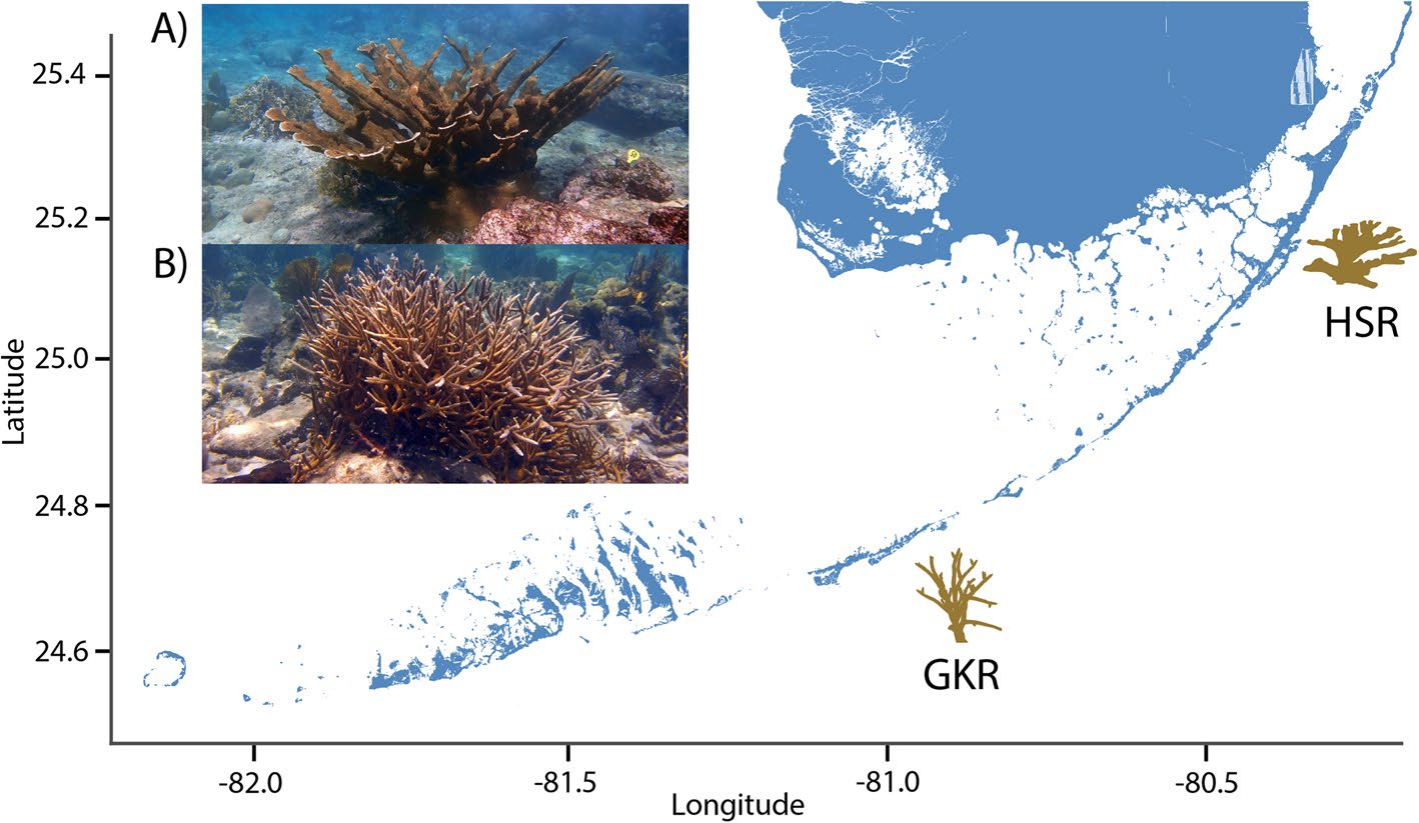
Sampling locations of *Acropora palmata* (**A**) and *A. cervicornis* (**B**). Both species are dominant reef-building corals of Caribbean and northwestern Atlantic reefs and are the only representative species of the genus *Acropora* in the region. Letter notation on the map indicates the geographic origin of *A. palmata* genome genet at Horseshoe Reef (HSR) and *A. cervicornis* genome genet near Grassy Key (GKR). Photos by IBB

Recombination allows for the separation of beneficial and detrimental alleles, such that selection may act upon them independently [52]. However, the role of recombination in adaptive evolution has been the subject of debate. While recombination has the capacity to create new, advantageous genetic combinations, it can also separate existing ones [53]. Recombination between adaptive loci may impede range expansions prompted by shifts in environmental conditions [54]. On the other hand, adaptive substitutions are correlated with higher recombination in several systems [8, 55, 56]. Further, recombination rate varies across individuals, across the genome, and across sexes [57, 58]. Global patterns of variation in recombination rates between males and females (heterochiasmy) across taxa suggest these differences may be adaptive [59]. Heterochiasmy in simultaneously hermaphroditic animals has been found in a limited number of studies published to date [60–62], and the recombination landscape of different sexes has only been studied in one other coral, *Acropora millepora* [60]. Here, we focus on the recombination landscape of two critically endangered sister species, *Acropora palmata* and *A. cervicornis* (Fig. 1). Both species are simultaneous hermaphrodites that reproduce sexually via gamete release and asexually via fragmentation [12]. Because these are endangered species, understanding their potential to adapt to changes is a pressing issue.

One way to derive recombination rates is by building a genetic linkage map. Linkage maps can be generated from just one cross with many offspring or from few offspring across several families [63]. Because one biparental coral cross can generate hundreds of offspring, many recombination events can be cataloged among siblings from a few families, or even a single family, and used to order markers along a chromosome. Using a combination of long read, short read, Hi-C chromatin scaffolding, and linkage map anchoring of de novo assembled scaffolds, we report chromosome-level genome assemblies and genetic maps of the two Atlantic acroporid species, *Acropora palmata* (Lamarck, 1816) and *A. cervicornis* (Lamarck, 1816). With these assemblies and maps, we compare patterns of macrosynteny and gene colinearity at the whole genome level with Pacific acroporids and distant relatives and characterize the recombination landscapes in these sister species.

## Methods

### Sample collection and sequencing

Adult coral tissue was collected from the *Acropora cervicornis* genet M5 collected near Grassy Key (24.711783° N, 80.945966° W) and reared at the Coral Restoration Foundation Tavernier Nursery (CRF, 24.9822° N, 80.4363° W) and the *A. palmata* genet HS1 from Horseshoe Reef (25.1399° N, 80.2946° W) (Supplementary Table 1; [47]). High molecular weight genomic DNA (gDNA) was isolated from each coral tissue sample using the Qiagen DNeasy kit (Qiagen, Valencia, CA) with slight modifications described previously [64]. Paired-end 250 bp sequencing libraries (avg. insert size 550 nt) were constructed from 1.8–2 µg gDNA with the TruSeq DNA PCR-Free kit (Illumina, San Diego, CA) and sequenced on the Illumina HiSeq 2500 by the Genomics Core Facility at Pennsylvania State University. Additionally, coral tissue from *A. palmata* HS1 was collected by CRF in January of 2018, snap-frozen in liquid nitrogen and sent directly to Dovetail Genomics for DNA extraction followed by Chicago and Hi-C library preparation.

For the PacBio libraries, gamete bundles of *A. cervicornis* M5 (spawned 2015 and August 22, 2016 at the CRF nursery) and *A. palmata* HS1 (spawned August 20, 2016 at Horseshoe Reef) were collected during the annual coral spawn. Once the gamete bundles broke apart, sperm was separated from the eggs using a 100 μm filter and concentrated and washed with 0.2 μm filtered seawater through three rounds of centrifugation at 2,000 × g for 5 min at room temperature. The *A. cervicornis* sperm samples from 2015 were brought to a final concentration of 3 × 10^7^ cells ml^−1^ after the addition of Cell Suspension Buffer and 2% agarose using the Bio-Rad CHEF Genomic DNA Plug Kits (Bio-Rad, Hercules, CA). Genomic DNA plugs were processed according to the manufacturer’s protocol and stored at 4 °C. The genomic DNA was extracted from the plugs in two ways, either using the QIAquick Gel Extraction kit (Qiagen) or by soaking the plugs overnight in 100 ul nuclease-free water at 4 °C followed by 1 h at -80 °C and recovered at 23,000 × g. Sperm samples of both species from 2016 were stored as 1 ml aliquots of concentrated sperm in 100% non-denatured ethanol at -20 °C until extraction. Genomic DNA was extracted using Nucleon Phytopure DNA extraction kit (Cytiva, Marlborough, MA) with the addition of RNase treatment and increased incubation time of 3 to 4 h at 65 °C during the cell lysis step. Genomic DNA elutions were combined and concentrated using the AMPure bead clean-up (final gDNA = 2 μg for *A. cervicornis* and 10 μg for *A. palmata*). Given the different final gDNA concentrations, PacBio libraries were prepared using a 20 kb size-selection protocol for *A. palmata* and a low input, no size selection protocol for *A. cervicornis*. Both libraries were sequenced on Sequel II by the Genomics Core Facility at Pennsylvania State University.

Because the initial *A. cervicornis* assembly exhibited low contiguity, an additional assembly was generated using Oxford Nanopore (ONT) long-read sequencing data. For the *A. cervicornis* ONT DNA library, coral tissue from the M5 genotype preserved in ethanol was provided by the Coral Restoration Foundation in 2021 and stored at -20 °C until extraction. Genomic DNA was extracted using the Qiagen MagAttract HMW DNA kit (MD, USA) following the manufacturer’s protocol. To further purify the gDNA, a salt-ethanol precipitation was performed. Briefly, 0.1 volumes of 3 M NaOAc (pH 5.2) were added to the DNA elution, followed by 3 volumes of 100% ethanol. The sample was centrifuged at approximately 20,000 × g for 1 h at 4 °C. The supernatant was then removed and the pellet was washed twice with cold 75% EtOH. The dried pellet was resuspended in Buffer AE (Qiagen, MD, USA) and long read libraries were generated using an Oxford Nanopore Ligation Sequencing Kit v10 (SQK-LSK110). Libraries were subsequently sequenced on an Oxford Nanopore PromethION flow cell (R9.4, FLO-PRO002) by the University of Wisconsin Biotechnology Center. Bases were called by the sequencing provider using Guppy v5.0.12 and delivered in fastq format.

### K-mer genome size estimation

We removed low-quality bases (Phred score below 25) and adaptors from Illumina reads, discarding reads shorter than 50 bp, with cutadapt v1.6 [65]. Prior to genome assembly, 119-mer counting was performed on trimmed reads from each sample using jellyfish v2.2.10 [66] for the purpose of haploid genome size estimation. We utilized 119-mers because a k-mer length of 119 was identified as the optimal k-mer for de novo genome assembly from contamination filtered reads by KmerGenie v1.7048 [67] after testing a range of k-mers from 21 to 121. K-mer frequency histograms were analyzed using the GenomeScope2 web portal [68] and findGSE [69], which use a negative binomial and skew distribution model, respectively.

### Contamination filtering of Illumina short read data

DNA extractions on the adult tissue used for Illumina sequencing were composed of the coral host and its associated microbial partners (algal symbionts and other microbes). To remove non-coral reads, we applied a modified series of filtering steps that compares sequence homology and GC content similar to process in BlobToolKit [70, 71] and described previously for *A. cervicornis* by Reich et al. [72]. Adaptor trimmed reads were initially assembled into contigs with SOAPdenovo2 v0.4 (parameters -K 95 –R) [73]. The contigs were compared to the genomes of the coral *Acropora digitifera* (NCBI: GCF_000222465.1; [74]), the symbiont *Breviolum minutum* (OIST: symbB.v1.0.genome.fa; [75]), and the NCBI nucleotide database (nt) using megablast (evalue 1e^−5^ threshold) [76]. Contigs with higher sequence similarity to non-cnidarians in the nt database were combined to make a local contamination database. Adaptor trimmed reads were then aligned with Bowtie2 v2.2.9 (parameters –q –fast; [77]) sequentially against the *A. digitifera* mitochondria (NBCI: KF448535.1), three concatenated Symbiodiniaceae genomes (*Symbiodinium microadriaticum, Breviolum minutum, Fugacium kawagutii*; [75, 78, 79], respectively) and the contamination database. Unaligned reads were extracted and used for short-read genome assembly described below.

### Hybrid genome assembly of* A. cervicornis *and *A. palmata*

The trimmed and filtered short reads were assembled with SoapDeNovo-127mer v2.04 [73] using different k-mers for each species, *A. palmata* K = 99 and *A. cervicornis* K = 95. Contigs were filtered for additional symbiont contamination using megablast against the three Symbiodiniaceae genome assemblies described above. A surprising number of symbiont contigs, roughly 500,000 in each species assembly, were present despite our read contamination filtering [72]. The non-symbiont contigs were then assembled with PacBio long reads using the hybrid method DBG2OLC [80], k = 17 MinLen = 500 AdaptiveTh = 0.001 KmerCovTh = 2 MinOverlap = 20). PacBio reads were also assembled separately with Canu v1.5 [81], genomeSize = 400 m correctedErrorRate = 0.075 minReadLength = 500). The two assemblies (hybrid and PacBio only) were then combined using QuickMerge v0.2 [82]*, A. palmata* = -hco 5.0 -c 1.5 -l 55000 -ml 1000; *A. cervicornis* = -hco 5.0 -c 1.5 -l 99500 -ml 1000) with the hybrid assembly as the reference and PacBio assembly as the query. Additional contig extension was performed with FinisherSC v2.1 [83]. Lastly, the assemblies were polished using Pilon v1.22 [84].

#### Hi-C scaffolding of hybrid *Acropora* palmata assembly

Our hybrid assembly of *A. palmata* was submitted to Dovetail Genomics for Hi-C analysis. They combined their proprietary HiRise scaffolding and Hi-C analysis (Supplementary Table 1), but the assembly was still far from chromosome-resolved (441 scaffolds, N_50_ = 6.8 Mb, and L_50_ = 16). In an effort to further improve the *A. palmata* genome assembly, we mapped the Hi-C paired-end reads separately back onto the Dovetail Genomics assembly with bwa-mem v 0.7.17 [85] with the mapping parameters -A1 -B4 -E50 -L0. We then followed the steps outlined by HiCExplorer v2.1.1 to create and correct a Hi-C contact matrix using default settings with a lower bin correction threshold of -1.5 [86]. This indicated there were more short range (< 20 kb) than long range (> 20 kb) contacts in the matrix. The corrected matrix was then used by HiCAssembler v1.1.1 [87] to further orient the scaffolds into pseudochromosomes with a minimum scaffold length set to 300,000 bp, a bin size of 15,000 and two iterations.

### Nanopore assembly of *Acropora* cervicornis

PromethION data was trimmed and filtered with Porechop [88], resulting in a total of 94 Gb across 39.91 M reads of usable ONT data. With trimmed ONT data, metaFlye [89] was used to perform a long-read only metagenome assembly. Following the initial metaFlye assembly, which includes a long-read polishing step, the assembly was further polished in one round using hypo [90]. Illumina short read data from the M5 genet described above was trimmed using TrimGalore [91], and mapped to the preliminary assembly with bwa-mem [85] prior to use with hypo. ONT reads were then mapped to the assembly using minimap2 [92] and BAM files were sorted using samtools [93]. Using blastn [94], assemblies were searched against a custom database comprised of NCBI’s ref_euk_rep_genomes, ref_prok_rep_genomes, ref_viroids_rep_genomes, and ref_viruses_rep_genomes databases combined with dinoflagellate and *Chlorella* genomes [75, 95–98]. Using the mapping and blastn hits files, blobtools [99] was used to identify and isolate cnidarian contigs. Purge_dups [100] was utilized to identify and remove any remaining putative haplotigs in the respective assembly.

### Linkage map construction

A full-sibling family was generated through a controlled cross between two *Acropora palmata* genets. Spawn was collected from two genets during the August 2018 spawning season in Curacao. Once egg-sperm bundles had broken apart, gametes were separated, and eggs were washed to remove any remaining self-sperm. The sperm from the genet designated as the sire was used to fertilize washed eggs from the genet designated as the dam. The resulting larvae were reared to 96 h post-fertilization in filtered seawater before preservation in individual 1.5 ml PCR tubes with 96% ethanol. A total of 105 full-sibling offspring were used in the construction of the genetic linkage map. Three to four polyps of each spawning parent were collected using coral cutters and preserved in 96% ethanol. For *Acropora cervicornis*, coral recruits from 16 families reared in a previous study until they first branched were used to construct a linkage map [15]. Samples of these recruits were preserved in 95% ethanol in 1.5 mL Eppendorf tubes and immediately placed into a -80° freezer until extraction.

For *Acropora palmata* larval offspring, high molecular weight DNA extractions followed the methods in Kitchen et al. [101]. Each larva was incubated in 12 μl of lysis solution (10.8 μl Buffer TL, 1 μl of Proteinase K, and 0.2 μl of 100 mg/ml RNAse A, all reagents from Omega BioTek) for 20 min at 55 °C. Next, 38 μl of Buffer TL and 50 μl of phenol/chloroform/isoamyl alcohol solution (25:24:1) was added to each sample and gently rocked for approximately 2 min. After centrifuging each sample for 10 min at 20,000 g, the top aqueous phase was removed and placed in a new tube. 50 μl of chloroform:isoamyl alcohol (24:1) was added to each sample and gently rocked for 2 min. Samples were centrifuged again at 10,000 rpm for 5 min and the top aqueous phase was again removed and placed into a new tube. The DNA was precipitated with 1.5 × volume of room-temperature isopropanol, 1/10 volume of 3 M sodium acetate (pH = 5.2) and 1 μl of glycogen (5 mg/ml) for 10 min at room temperature. Samples were then centrifuged at 20,000 g for 20 min and washed with 70% ice-cold ethanol. All supernatant was removed, and pellets were dried under a hood for approximately 30 min. Pellets were re-suspended in 30 μl of low TE buffer (10 mM Tris–HCl and 0.1 mM EDTA). Parental tissue was extracted using Qiagen DNeasy kit (Qiagen, Valencia, CA) following the modified protocol described in Kitchen et al. [101] and eluted in 100 μl of nuclease-free water.

Extracted samples were genotyped using the Applied Biosystems Axiom Coral Genotyping Array—550,962 (Thermo Fisher, Santa Clarita, CA, USA). The raw data were analyzed using the Axiom ‘Best Practices Workflow’ (BPW) with default settings (sample Dish QC ≥ 0.82, plate QC call rate ≥ 97; SNP call-rate cutoff ≥ 97; percentage of passing samples ≥ 95). The resulting genotyping files were converted to variant caller format (VCF) using the bcftools plugin affy2vcf [102] and filtered to represent only the recommended probeset identified by the Axiom BPW.

*Acropora cervicornis* recruits were sampled from the base of the colonies and DNA was extracted by Eurofins BioDiagnostics (WI, U.S.A) using LGC (Hoddesdon, UK) Sbeadex Animal DNA Purification Kits. Samples were run on two plates of the Applied Biosystems Axiom Coral Genotyping Array. *Acropora cervicornis* cross data was processed in the same manner as *A. palmata*, using the Axiom workflow and subsetting single nucleotide variants to only include recommended probes.

*Acropora palmata* and *A. cervicornis* linkage analysis was carried out using Lep-MAP3 [63] using the wrapper pipeline LepWrap [103]. Markers were first filtered for deviation from Mendelian inheritance and missing data via the Lep-MAP3 module ParentCall2. For *A. cervicornis*, the flag halfSibs = 1 was added to ParentCall2 to account for shared parentage among crosses. Recombination informative markers (here defined as those that were heterozygous in at least one parent) were next filtered using the Filtering2 module with a data tolerance of 0.0001. The remaining markers were assigned to 14 linkage groups (LGs) using an LG minimal size limit set to 5 markers using the module SeperateChromosomes2 and a logarithm of odds (LOD) score of 11 in *A. palmata* and 5 in *A. cervicornis*. For *A. palmata*, an informativeMask value of “123” was used and for *A. cervicornis* multi-family data, an informativeMask of “12” was used. Unassigned markers were iteratively added to existing LGs using a LOD limit of 2 and a LOD difference of 2. Markers were next ordered using the Kosambi mapping function as implemented in the module OrderMarkers2 with the identical limit set to 0.005, usePhysical = 1 0.1, 100 merge iterations, 3 phasing iterations, and the hyperPhaser parameter used to improve marker phasing. To remove markers at map edges that may erroneously inflate the map length, the last 10% of markers were trimmed if they fell more than 5% of the total centimorgan (cM) span away from the next nearest marker. After trimming, marker order was evaluated with a second round of OrderMarkers2 using the same parameters as previously described. Both paternal and maternal maps were generated and the option sexAverage = 1 was applied to include a sex-averaged consensus map. Average marker distance was calculated as the size of the linkage map in cM divided by the number of markers. As global orientation of a linkage group is arbitrary in Lep-MAP3, marker order was flipped for LGs in which the start of the genetic map (0 cM) corresponded to the end, rather than to the start of the physical map (the position 0 bp) of a given scaffold. To generate cleaned Marey maps, MareyMap Online [104] was used to remove aberrant markers and generate smoothed recombination maps using 2-degree polynomial LOESS estimation with a span of 0.25.

### Linkage scaffolding of* A. cervicornis* Nanopore assembly

For *A. cervicornis*, no Hi-C data was available. As such, the *A. cervicornis* assembly was scaffolded using Lep-Anchor [105] with the linkage map generated by Lep-MAP3 [63]. To assist in orientation of contigs with markers, as well as placements of contigs without markers, minimap2 v2.24 [92] was used to generate a PAF file using the ONT data. Lep-Anchor was run via LepWrap and utilized default Lep-Anchor arguments, apart from setting the expected number of linkage groups to 14. Additionally, LepWrap implements the edge-trimming scripts for Lep-Anchor as was described above for Lep-MAP3.

### Repeat identification, masking, and divergence analysis

For both assemblies, repetitive sequences were predicted with RepeatModeler v 1.0.11 [106], filtered for genuine genes based on blast similarity to the NCBI nr database or *Acropora digitifera* protein sequences (*e*-value ≤ 1e^−5^), combined with the *Acropora* TE consensus sequences in Repbase (*n* = 149), annotated separately against the invertebrate repeat database in CENSOR v4.2.29 [107] for “unknown” TEs, and soft masked using RepeatMasker v 4.0.7 [108]. We also ran the above series of steps on the genome assemblies of *A. digitifera*, *A. tenuis* and *A. millepora* to ensure comparable repeat estimates. The summary table for each species was generated using the buildSummary.pl utility script, and TE accumulation was calculated as the Kimura substitution level corrected for CpG content from the respective consensus sequence produced using the calcDivergence.pl and createRepeatLandscape.pl utility scripts in RepeatMasker. Kimura distance was converted to Jukes-Cantor distance using the formula JC = − 3/4*log(1 − 4*d/3), where *d* is the distance estimated by RepeatMasker. Assembly-free repeat identification, annotation and quantification was performed on 25% of the adapter-trimmed Illumina short-read data of each Atlantic species using dnaPipeTE v1.3.1 [109].

### Gene prediction and annotation

For the *A. palmata* assembly, we used a combination of ab initio (GeneMark-ES v4.32; [110]) and reference-based tools (BRAKER v2.0; [111], PASA v2.1.0; [112], and exonerate v2.2.0; [113]) for gene prediction as previously described [114]. For BRAKER, RNAseq data produced on the Roche 454 GS FLX Titanium system was obtained from NCBI Bioproject PRJNA67695 [115] and mapped to the assembly using STARlong v2.5.3a [116] due to the average read lengths being greater than 300 bp. Gene models with read coverage greater than or equal to 90% were assigned as “BRAKER_HiQ'' predictions. The assembled *A. palmata* transcriptome from Polato et al. [115] was used as the input for PASA. Homology-based gene predictions were made with exonerate against all eukaryotic sequences in the UniProt database (*n* = 186,759), keeping predictions with at least 80% coverage. Gene predictions were combined with EVidenceModeler [112]. We also predicted tRNA sequences using tRNAscan_SE v1.3.1 [117]. The predicted genes were searched against the NCBI nr, UniProt Swiss-Prot and Trembl databases, and KEGG Automated Annotation Server. Blast-based searches were filtered by the top hit (*e*-value < 1e-5 threshold). GO annotations were extracted from UniProt of NCBI databases. Genes were also compared to OrthoDB v10.1 [118]. Gene annotation was assigned based on the *e*-value score < 1e-10 first to Swiss-Prot followed by Trembl and then NCBI. If no sequence homology was recovered, then the gene was annotated as a “hypothetical protein”. Gene predictions from the hybrid assembly were lifted over to the final Hi-C assembly using the UCSC liftOver process [119]. We also used homology-based prediction tool GeMoMA v1.6.1 [120] to map the *A. palmata* gene models to the Hi-C assembly. Liftover and GeMoMa predictions were combined with EVidenceModeler for the final gene set.

The original PacBio *A. cervicornis* assembly was annotated in a similar manner to *A. palmata*. However, the original assembly is superseded here by the ONT-based assembly. The ONT *A. cervicornis* LepWrap*-*scaffolded assembly was annotated using funannotate v1.8.13 [121] with RNAseq data obtained from four BioProjects available on NCBI SRA at the time of assembly (PRJNA222758, PRJNA423227, PRJNA529713, and PRJNA911752). All RNAseq data was adapter- and quality-trimmed using TrimGalore [91]*.* Briefly, funannotate train was run with a –max_intronlen of 100,000. Funannotate train is a wrapper that utilizes Trinity [122] and PASA [112] for transcript assembly. Upon completion of training, funannotate predict was run to generate initial gene predictions using the arguments –repeats2evm, –organism other, –max_intronlen 100,000, and –repeat_filter none. Additional transcript evidence from three sources (the initial *A. cervicornis* annotation described above, transcripts from Selwyn and Vollmer, [123], and the Osborne transcriptome, [124]) was provided to funannotate predict using the –transcript_evidence argument. Funannotate predict is a wrapper intended to separately run AUGUSTUS [125] and GeneMark [110] for gene prediction and EVidenceModeler [112] to combine gene models. Funannotate update was run to update annotations to be in compliance with NCBI formatting. For problematic gene models, funannotate fix was run to drop problematic IDs from the annotations. Finally, functional annotation was performed using funannotate annotate which annotates proteins using PFAM [126], InterPro [127], EggNog [128], UniProtKB [129], MEROPS [130], CAZyme [131], and GO [132].

#### Whole genome alignments and gene-level divergence

Genome assemblies of *A. palmata*, *A. cervicornis* M5 genet, and *A. cervicornis* K2 genet were aligned using minimap2 [92] with “asm5” setting for whole genome alignments, and the nucmer command within the mummer v4.0 package [133] with a minimum exact match length of 100 bp (-l 100), minimum cluster length of 500 (-c 500) and using all anchor positions (–maxmatch). To assess genome-scale synteny, the PAF alignments from minimap2 were plotted using both R package pafr v0.0.2 [134] and dotplotly [135]. The delta alignments from *nucmer* were visualized using the D-Genies web server [136]. Structural variants (insertions, deletions, tandem duplications and contractions, inversions and translocations) were identified from the whole genome alignments of *A. palmata* and *A. cervicornis* M5 genet using three tools: assemblytics [137], MUM&Co [138], and SVIM-asm [139]. Only MUM&Co and SVIM-asm were able to detect inversions and translocations.

To assess sequence divergence between the two species at the gene-level, rustybam [140] was used to split PAF alignments at gene coordinates and to calculate gene-level percent identity using matches and mismatches. Genes were considered outliers in sequence divergence if their percent identity was less the first quartile (Q1) minus three times the interquartile range (IQR). Enrichment analyses were performed using clusterProfiler v4.4.4 [141] with a custom database for *A. palmata* created with AnnotationForge v1.38.0 [142] to test for enrichment of gene ontology (GO) terms in the outlier gene set.

#### Orthologous gene identification and macrosynteny analysis

Genome completeness of each acroporid assembly was assessed using BUSCO v4.1.1 with the Metazoa odb10 orthologous gene set (*n* = 954 orthologues, [143]). To discover shared and unique gene families in *A. cervicornis* and *A. palmata* in relation to other species, OrthoFinder v2.5.2 [144] was run on the predicted proteins of each species listed in Supplementary Table 2 The species tree was constructed from a multiple sequence alignment with STAG and rooted by STRIDE in OrthoFinder v2.5.2 [144]. A presence/absence table of orthogroups, or sets of genes descended from a single gene in the last common ancestor of all the species being considered, was used to generate an UpSet intersecting set plot ( [145], as implemented in UpSetPlot [146]. The species tree from OrthoFinder was time-calibrated using r8s [147] with priors for *Acropora* (101 million years, [148]), Acroporidae (168 million years, [149]), Scleractinia (268 million years, [150]), and Anthozoa (541 million years, [151]) accessed via the Paleobiology Database [152]. CAFE 5 [153] was used to analyze time-calibrated phylogenies and the phylogenetic hierarchical orthogroups from OrthoFinder to identify gene families undergoing significant expansion or contraction in each node and tip. For nodes relevant to this study (Acroporidae, *Acropora,* Atlantic *Acropora*), we extracted significantly changing phylogenetic hierarchical orthogroups, as well as standard orthogroups unique to relevant nodes, and performed GO enrichment tests with clusterProfiler v4.4.4 [141] using a custom database for *A. palmata* created with AnnotationForge v1.38.0 [142].

Macrosyntenic patterns across the species with chromosome-resolved genome assemblies was assessed with Oxford Dot Plots (ODP, [154]), specifically mapping on the inferred ancestral linkage groups (ALGs) of sponge, cnidarian and bilaterians recently identified [2]. ODP runs an all-vs-all blast akin to OrthoFinder with diamond v2.0.15 [155] and identifies conserved syntenic gene arrangements between two genomes. Dot plots and ribbon diagrams were generated by ODP with default settings and restricting plotted scaffold length of 2 Mb to visualize conserved syntenic blocks across closely related or more distant taxa.

## Results

### Chromosome-scale genome assemblies of the Atlantic acroporids

**Fig. 2:**
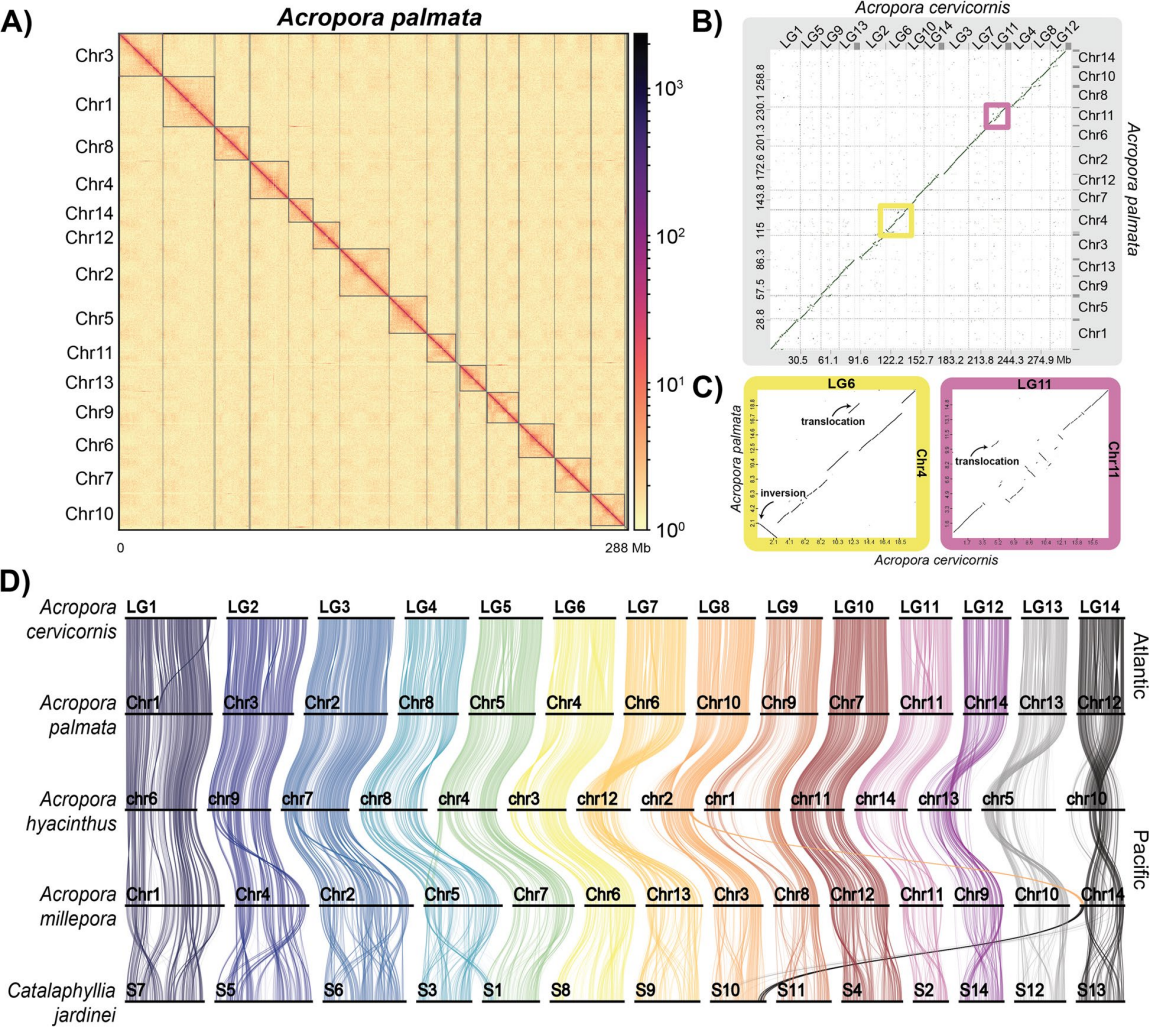
Atlantic acroporid genome assemblies. **A** Hi-C contact map of *A. palmata* genome resolved into 14 chromosomes using HiCAssembler [87]. **B** Dot plot visualization of colinear relationships of the 14 chromosomes/linkage groups between the sister species *A. palmata* (y-axis) and *A. cervicornis* (x-axis) using the D-genies web server [136]. The scale on each axis is in megabases (Mb). The points along the diagonal represent colinear genomic regions whereas those dots off the diagonal represent rearrangements (insertions, deletions, inversions and translocations). Yellow and purple boxes highlight two chromosomes, *A. cervicornis* LG6 and LG11, with complex rearrangements. (**C**, left) Comparison of *A. cervicornis* LG6 to *A. palmata* Chr4 reveals a 2.5 Mb inversion and 1.4 Mb translocation. (**C**, right) Complex rearrangements observed between *A. cervicornis* LG11 and *A. palmata* Chr 11, including a 0.765 Mb translocation. **D** Ribbon plot of syntenic orthologous genes conserved among scleractinians. The colored vertical links connect orthologous genes to the numbered chromosomes of the five species, represented by horizontal bars. Chromosome fusions or fissions are represented by crossing over of the colors that represent each ancestral linkage group. Chromosomal inversions were detected between Atlantic and Pacific acroporids (e.g. *A. cervicorni*s L4, L6, and L12). Chromosomal changes were more numerous between Pacific than Atlantic acroporids. Comparing *A. hyacinthus* Chr 5, 10, 12 and 13 to all other acroporids indicates paracentric inversions of whole chromosome arms in this species

To investigate the genomic conservation and divergence between the two Atlantic acroporids, we generated chromosome-scale genome assemblies for both species collected from the Florida Keys. For *A. palmata* (genet HS1, STAGdb ID HG0004), we used a hybrid assembly strategy that combined PacBio Sequel II long-reads with Illumina paired-end short reads to obtain an initial assembly with 2,043 scaffolds totaling to 304 Mb and an N_50_ of 282 kb (N_50_ is the minimum contig length to cover 50% of the genome). The assembly was further improved with Dovetail Chicago HiRise and Dovetail Hi-C data (all data used for genome assembly in both species described in Supplementary Table 1). After Hi-C scaffolding, the final 287 Mb haploid assembly was resolved into 14 pseudochromosomes (hereafter referred to as chromosomes, labeled Chr1—Chr14), a number consistent with the karyotype of *A. palmata* [156]. The *A. palmata* assembly has 406 scaffolds with an N_50_ of 18.66 Mb (Fig. 2A and Supplementary Table 3).

For *A. cervicornis* (genet M5, STAGdb ID HG0005), we initially used the same hybrid assembly strategy as for *A. palmata* relying on a combination of PacBio Sequel and Illumina short-read data (Supplementary Table 1). However, due to reduced high molecular weight genomic DNA available at the time, we were unable to size-select our PacBio library as we did for *A. palmata*, yielding shorter read lengths with an average read length and N_50_ of 3,238 bp and 4,394 bp, respectively, compared to 7,126 bp and 10,110 bp in *A. palmata* (Supplementary Table 1). Our first assembly was consequently less contiguous, with 4,382 scaffolds in 318 Mb and an N_50_ of 162 kb. We next turned to Oxford Nanopore PromethION (ONT) sequencing to generate additional long-read sequences but due to sample quality, the run produced an average read length of 2,366 bp, albeit with much higher overall data yield of 94.4 Gbp. Assembly of the high coverage ONT reads resulted in 6,381 contigs with an N_50_ of 711 Kb. To further resolve the *A. cervicornis* genome, we constructed a linkage map (described below) that was used to anchor and orient the ONT contigs into 14 linkage groups (LGs). These LGs correspond with high synteny to the Hi-C chromosomes assembled for *A. palmata*. Thus, the *A. cervicornis* LGs can be considered (pseudo)chromosomes. To better distinguish chromosomes for each species, we number the *A. cervicornis* chromosomes here as LG1—LG14. The final 305 Mb assembly was slightly more contiguous than *A. palmata* with a scaffold N_50_ of 20.05 Mb.

Our assemblies of *A. palmata* (287.6 Mb) and *A. cervicornis* (305.4 Mb) were on the lower end of the predicted genome sizes from three different k-mer based tools that ranged from 290 to 354 Mb (Supplementary Table 4), and both assemblies are approximately 110 to 180 Mb smaller than genomes of other acroporids species assembled to date (Supplementary Table 3). When comparing estimates of genome completeness using BUSCO Metazoa v10 [143, 157], we identified 87.8% complete genes in *A. palmata*, compared with 93.1% in *A. cervicornis* (Supplementary Table 5). Both assemblies exhibit minimal remaining haplotig duplication, with 1.2% of BUSCO genes in *A. palmata* duplicated and 0.3% in *A. cervicornis*.

Recently, a genome assembly of another *A. cervicornis* genotype from the Florida Keys, genet K2 (STAGdb ID HG0582), was published [123]. Using minimap2 [92] whole genome alignments, we demonstrate that the two assemblies are mostly concordant (Supplementary Fig. 1). Both assemblies are similar in completeness according to BUSCO Metazoa v10 [143, 157] assessment with the M5 assembly (this study) showing 93.1% completeness and the K2 assembly showing 92.45% completeness, of which 0.30% and 0.42% are duplicated, respectively (Supplementary Table 5). The assemblies are similar in size, with the M5 assembly being 305 Mb in total length and the K2 assembly 307 Mb. The most notable difference is the gain in scaffold length, with a scaffold N50 of 20.051 Mb for the M5 assembly, compared with 2.8 Mb for the K2 assembly. The K2 assembly has more contiguous primary contigs, with a contig N50 of 2.711 Mb compared with 0.732 Mb in the M5 assembly. Some K2 contigs are split across multiple linkage groups in the M5 assembly (Supplementary Fig. 1). These regions may reflect novel structural variants between genets within the Florida population of *Acropora cervicornis* or may represent misassembly in either assembly. Hi-C scaffolding or additional ultra-long read sequencing should be performed to validate the structural variants between these *A. cervicornis* assemblies.

### Genomic synteny is largely conserved in the sister species

Whole genome alignments of the two Atlantic acroporid genomes using minimap2 [92] and nucmer [133] revealed long stretches of colinear regions with interspersed rearrangements across the 14 chromosomes (Fig. 2B). As well as similarities, there were differences in physical lengths of chromosomes that resulted in different chromosome number/linkage group naming assignments for each species (see Supplementary Table 6). For example, the length of the corresponding syntenic chromosome pair of *A. cervicornis* LG2 was 4.87 Mb longer than *A. palmata* Chr3. Overall, we identified 10,532 structural variants (SV) totaling 33.02 Mb between the two assemblies using variant calling tools (Supplementary Table 7). An additional 1.4 Mb translocation was detected by whole genome alignment dot plots between *A. cervicornis* LG6 and *A. palmata* chromosome Chr4 (Fig. 2B and C). Dot plots also highlighted a large inversion of 2.5 Mb between the same syntenic chromosome pair (*A. cervicornis* LG6 and *A. palmata* Chr4) and numerous smaller SV types were identified near the middle of *A. cervicornis* LG11 and *A. palmata* Chr11 (Fig. 2C), a region that may correspond with the centromere.

At the gene level, percent identity between the two species is high, with orthologs maintaining a median nucleotide percent identity of 99.65%. A total of 536 genes exceeded the Q1-(3*IQR) threshold to be considered an outlier gene in terms of percent identity (Supplementary Table 8). These outlier genes exhibited no enrichment of molecular function or biological process GO terms but showed enrichment at two cellular process GO terms: spindle pole (GO:0000922, *p.adjust* = 0.03) and midbody (GO:0030406, *p.adjust* = 0.04). The one annotated gene with the lowest percent identity (83.83%) that is not associated with transposable elements is a homolog of the sulfatase-modifying factor 1 (SUMF1). Other notable outlier genes include genes associated with gamete compatibility and fertilization (SPAG1 and REJ).

### Synteny and gene content in the genus *Acropora*

We then compared the genome architecture and gene content of the Caribbean acroporids to other acroporids. To predict gene models for each assembly, we used a combination of transcriptomic data and ab initio tools resulting in 31,827 and 34,013 genes in *A. palmata* and *A. cervicornis*, respectively (Supplementary Table 3). Combining our gene models with those of other acroporids with chromosome-resolved assemblies, we identified colinear (shared loci with the same arrangement on a given chromosome) and macrosyntenic (shared loci not necessarily in the same arrangement on a given chromosome) gene arrangements (Fig. 2D and Supplementary Fig. 2A). In accordance with the high degree of synteny at the whole genome level, 15,873 out of 17,243 one-to-one orthologs between *A. palmata* and *A. cervicornis* retained their colinearity (Supplementary Fig. 2). The number of orthologs that shared ordinal positions between *A. cervicornis* chromosomes and *A. hyacinthus* or *A. millepora* was 12,603 out of 13,000 and 12,075 out of 14,738, respectively. We found that the architecture of some chromosomes was largely unchanged at this scale of observation (e.g. *A. cervicornis* LG1 across acroporids, Fig. 2D). Nevertheless, several translocations and inversions were evident. Within the acroporids, interchromosomal translocations were observed in *A. millepora* with 85 genes of *A. cervicornis* LG8 located on Chr 14 of *A. millepora* and 132 genes of *A. cervicornis* LG5 located on *A. millepora* Chr 5 (Fig. 2D). Paracentric inversions of whole chromosome arms likely led to the *A. hyacinthus* Chrs 5, 10, 12 and 13 arrangements (Fig. 2D and Supplementary Fig. 2A).

### Most scleractinians share six of 21 cnidarian ancestral linkage groups

Ancestral chromosomal fusions and rearrangements within the coral lineage were detected by mapping previously inferred ancestral linkage groups (ALGs) shared among sponges, cnidarians and bilaterians against our genomes [2]. We note changes in ancestral ALGs in the reporting of results below with fusions represented by the letter “x” (Supplementary Table 9). Of the 21 cnidarian- specific ALG arrangements, six (*A1a, Ea, J1xQa, A1bxB3, NxA2,* and *B1xB2*) were largely intact within the scleractinians (acroporids and *Catalaphyllia*), represented by LG7, LG13, LG12, LG11, LG14 and LG5 in *A. cervicornis* (Supplementary Fig. 2B and Supplementary Table 9). Interestingly, ALG *Qb* was lost from all cnidarian species surveyed here, with the exception of the jellyfish *Cassiopea xamachana* that largely retains the ancestral cnidarian ALG structure (Supplementary Table 9 and Supplementary Fig. 3). We identified seven cases of ALG fusions and one example of centric insertion within one of the acroporid chromosomes, represented by *A. cervicornis* LG10 (Supplementary Fig. 2B and Supplementary Table 9). *A. millepora* is the only acroporid species where a portion of ALG *G* fused with *L*.

### Orthogroup analysis reveals gene family expansions in the acroporids

**Fig. 3:**
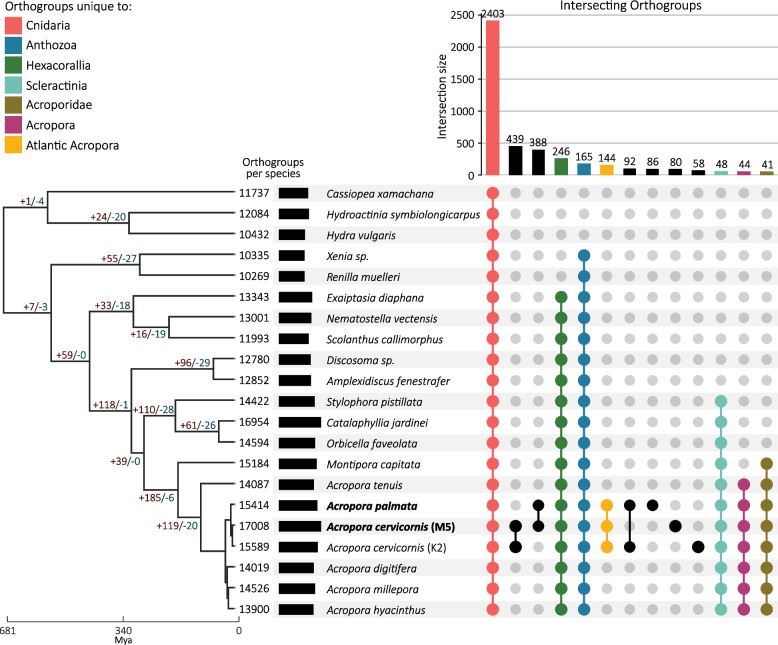
Conservation of gene content among cnidarians. UpSet plot displaying the number of shared orthologous groups amongst selected taxonomic groups—Cnidaria (red), Anthozoa (blue), Hexacorallia (green), Scleractinia (teal), Acroporidae (green brown), *Acropora* (purple) and Atlantic (Caribbean) *Acropora* (yellow). The colored or black circles below the vertical bar chart indicate those species that belong to each intersection group. On the left, the bar chart represents the total number of orthologous groups identified in each taxon. Taxon labels in bold were assembled in this study. The species tree constructed from a multiple-sequence alignment of 1,011 single-copy orthogroups (348,712 amino acid positions) was inferred by STAG and rooted by STRIDE in OrthoFinder v2.5.2 [144]. The species tree was time-calibrated using r8s [147] with priors for *Acropora* (101 million years, [148]), Acroporidae (168 million years, [149]), Scleractinia (268 million years, [150]), and Anthozoa (541 million years, [151]) accessed via the Paleobiology Database [152]. Node values depict the number of significant (*p* < 0.05) gene family expansions ( +) and contractions (-) identified by CAFE 5 [153]. Node values are not depicted for nodes internal to *Acropora*

Expanding beyond the species with chromosome-resolved assemblies*,* we compared orthologous gene families, also known as orthogroups, shared among diverse cnidarian taxa, including representatives of the Hexacorallia and Octocorallia within Anthozoa and Hydrozoa and Scyphozoa within Medusozoa (Supplementary Table 2). We identified 2,403 conserved orthogroups among all cnidarians (Fig. 3). There are 165 unique orthogroups in Anthozoa enriched in the process angiogenesis (GO:0001525, *p.adjust* = 0.049) and 48 unique Scleractinia orthogroups enriched in growth factor binding (GO:0019838, *p.adjus*t = 0.009), cell adhesion molecule binding (GO:0050839, *p.adjust* = 0.035) and D-inositol-3-phosphate glycosyltransferase activity (GO:0102710, *p.adjust* = 0.008). We further found 44 and 144 unique orthogroups in acroporids and Atlantic acroporids, respectively (Fig. 3). Only 39 of the 144 orthogroups shared between the Atlantic species were annotated, 12 of which were predicted as transposable elements.

In addition to orthogroups unique to each clade, CAFE 5 [153] was used to identify shared phylogenetic hierarchical orthogroups undergoing significant expansion or contraction within each node and tip of the species tree. We found a total of 191 gene families undergoing a significant change at the node associated with the family Acroporidae (+ 185 expanding and -6 contracting), 139 in the genus *Acropora* (+ 119 and -20), and 434 in the Atlantic *Acropora* (+ 247 and -187) (Fig. 3). Within the significantly changing gene families of Acroporidae, there were predominantly expansions, with notable GO enrichment in transcription-related terms, such as the PRC1 and ASAP complexes (GO:0035102; *p.adjust* = 1.78e-18 and GO:0061574; *p.adjust* = 4.59e-08), transposition terms (GO:0032196; *p.adjust* = 6.82e-29, GO:0006313; *p.adjust* = 1.85e-19), and symbiont-containing vacuole membranes (GO:0020005; *p.adjust* = 1.53e-08, Supplementary Fig. 4).

Within the genus *Acropora*, gene families primarily expanded (Fig. 3), with GO enrichment highlighting terms associated with cell–cell adhesion (GO:0098609; *p.adjust* = 6.98e-05), cell recognition (GO:0008037; *p.adjust* = 5.92e-16), and nerve components (e.g. myelin sheath and potassium channel complex, GO:0043209; *p.adjust* = 2.45e-10 and GO:0034705; *p.adjust* = 2.81e-08, respectively) and processes (e.g. paranodal junction assembly, GO:0030913; *p.adjust* = 7.52e-21, Supplementary Fig. 5). Finally, Atlantic *Acropora* exhibit enrichment related to transcriptional complexes (PRC1 complex, GO:0035102; *p.adjust* = 2.11e-06), as well as terms related to transposition (GO:0032196; *p.adjust* = 1.00e-88 and GO:0006313; *p.adjust* = 7.51e-70), retrotransposition (GO:0044826; *p.adjust* = 1.18e-07), and viral response (e.g. viral capsid, GO:0019028; *p.adjust* = 9.27e-03, Supplementary Fig. 6).

### Repetitive content is comparable among acroporids

Repetitive DNA plays a significant role in the size, organization and architecture of eukaryotic genomes [158]. To analyze transposable element (TE) content among the acroporid genome assemblies, we constructed species-specific repeat libraries for each assembly using a genome-guided approach with RepeatModeler [106]. To ensure that only bona fide repeats were included in our comparisons, we filtered out putative genes using a sequence similarity approach against the NCBI protein database or *A. digitifera* gene models. Using RepeatModeler and RepeatMasker, our analyses found a TE content of 16.69% in *A. palmata* and 18.91% in *A. cervicornis* (Supplementary Table 10). Using dnaPipeTE [109], an assembly-free method based on the Illumina short-reads, the total TE content was estimated to be 37.11% for *A. palmata* and 35.54% for *A. cervicornis* (Supplementary Fig. 7).

**Fig. 4:**
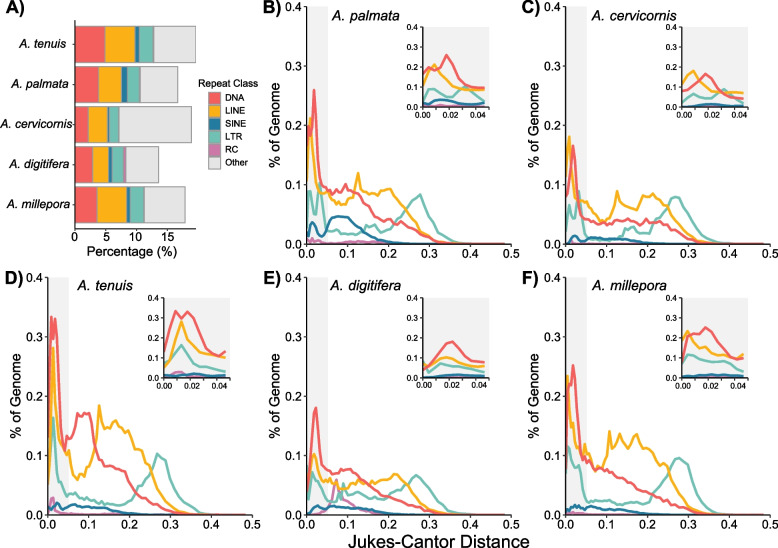
Comparison of repetitive DNA among acroporid taxa. (A) Percentage of the genome attributed to the main transposable element classes [DNA transposons, long interspersed nuclear element (LINE), short interspersed nuclear element (SINE), long terminal repeat (LTR), rolling circle (RC) and other (satellites, simple repeats, and unclassified)] for each acroporid taxon. (**B-F**) Repeat landscapes of all transposable element classes except “other” for *A. palmata* (**B**), *A. cervicornis* (**C**), *A. tenuis* (**D**), *A. digitifera* (**E**) and *A. millepora* (**F**). The percentage of genome coverage (y-axis) of each repeat is shown relative to the Jukes-Cantor genetic distance observed between a given repetitive element and its respective consensus sequence. Individual repetitive elements were then summarized by their repeat class. The more recent repetitive element copies have lower Jukes-Cantor distance on the left side of the x-axis. The inset plot in each panel focuses on recent repeat insertions at a Jukes-Cantor distance below 0.05 (gray shaded region in full plot)

The dominant TEs were shared among the species we surveyed across methods. These TEs belong to DNA transposons superfamilies Tc/Mariner and hAT, long interspersed nuclear element (LINE) retrotransposon family Penelope and long terminal repeat (LTR) family Gypsy (Supplementary Table 10). The transposable activity of each repeat class was compared across species to determine if TE accumulation differed over their evolutionary past (Fig. 4B-F). Each species experienced a recent burst of DNA, LINE and LTR copies in their genomes, as evidenced by the increased genomic coverage of those classes with zero to very small genetic distances (Fig. 4B-F inset plots). Within the recent TE expansion, the Atlantic acroporids and *A. millepora* have a bimodal distribution of LTR transpositions, specifically those within the retrotransposon family Gypsy. Overall, however, few species-specific patterns emerged in the repeat landscapes of the acroporids.

### Genetic Maps

In *A. palmata* we assigned 2,114 informative markers to 14 linkage groups (LGs), representing the 14 chromosomes, with an average marker distance of 0.48 cM and a consensus, sex-average map length of 1,013.42 cM (Table 1). The gamete-specific maps exhibited heterochiasmy and varied in length, with a longer female map length (1,460.68 cM) than male map length (583.19 cM). At the chromosome-level, female map lengths were longer than male map lengths in all 14 chromosomes (Table 1). The genome-wide average recombination rate was higher in the female (5.49 cM/Mb) than in the male (2.19 cM/Mb) map (Table 1). The highest average recombination rate (7.00 cM/Mb) was in the female map associated with Chr11. The lowest average recombination rate (1.55 cM/Mb) was in the male map associated with Chr2.

**Table 1:** Genetic map summary statistics for *Acropora palmata* and *Acropora cervicornis*. Physical lengths, map length, and average recombination rates per chromosome for male, female, and sex-averaged maps of *Acropora palmata* and *A. cervicornis*. Mb = megabases, cM = centimorgan

	Chromosome	Length (Mb)	Number of Markers	Male Map Length (cM)	Female Map Length (cM)	Sex Averaged Map Length (cM)	Male Recombination Rate (cM/Mb)	Female Recombination Rate (cM/Mb)	Sex Averaged Recombination Rate (cM/Mb)
***Acropora palmata***	Chr1	27.05	318	54.05	148.29	100.3	2	5.48	3.71
	Chr2	25.92	171	40.18	109.24	74.4	1.55	4.21	2.87
	Chr3	21.9	155	42.18	116.88	79.18	1.93	5.34	3.62
	Chr4	20.87	162	39.21	106.44	72.52	1.88	5.1	3.47
	Chr5	20.54	162	36.65	95.01	65.39	1.78	4.63	3.18
	Chr6	19.02	141	30.51	82.65	56.32	1.6	4.35	2.96
	Chr7	18.66	143	30.61	102.66	66.01	1.64	5.5	3.54
	Chr8	18.59	134	52.66	124.59	88.27	2.83	6.7	4.75
	Chr9	17.67	129	47.71	96.2	71.22	2.7	5.44	4.03
	Chr10	16.55	142	27.67	92.01	59.61	1.67	5.56	3.6
	Chr11	16.42	113	51.94	115.02	81.74	3.16	7	4.98
	Chr12	14.67	150	59.69	97.83	77.91	4.07	6.67	5.31
	Chr13	14.61	112	28.92	79.95	53.63	1.98	5.47	3.67
	Chr14	13.63	82	41.22	93.92	66.93	3.02	6.89	4.91
***Acropora cervicornis***	LG1	30.19	442	73.09	121.41	97.25	2.42	4.02	3.22
	LG2	26.77	495	50.62	96.61	73.61	1.89	3.61	2.75
	LG3	25.26	356	46.01	90.6	68.3	1.82	3.59	2.7
	LG4	20.97	433	54.48	89.59	72.03	2.6	4.27	3.44
	LG5	20.93	562	53.8	108.28	81.04	2.57	5.17	3.87
	LG6	20.56	340	27.42	76.07	51.75	1.33	3.7	2.52
	LG7	20.05	305	33.67	87.65	60.66	1.68	4.37	3.03
	LG8	18.96	310	44.65	94.95	69.8	2.36	5.01	3.68
	LG9	18.53	340	48.51	78.94	63.73	2.62	4.26	3.44
	LG10	18.31	256	39.53	88.94	64.24	2.16	4.86	3.51
	LG11	17.26	170	19.07	61.6	40.34	1.1	3.57	2.34
	LG12	15.82	231	43.29	81.06	62.17	2.74	5.12	3.93
	LG13	15.29	277	20.43	71.73	46.08	1.34	4.69	3.01
	LG14	14.96	342	47.38	105.34	76.36	3.17	7.04	5.1

The *A. cervicornis* linkage map was constructed with more offspring (154) from 16 families, and thus a greater number of informative markers were utilized in generating a consensus linkage map. In total, 4,859 markers were assigned to 14 linkage groups (LGs), with an average marker distance of 0.19 cM and a consensus map length of 927.36 cM (Table 1). Maps of *A. cervicornis* echoed the heterochiasmic maps of *A. palmata*, with a longer female map length (1,252.78 cM) than male map length (601.93 cM). As in *A. palmata,* for all 14 *A. cervicornis* LGs the female length was longer than the male length (Table 1). The genome-wide average recombination rate was higher in the female (4.41 cM/Mb) than in the male map (2.12 cM/Mb) (Table 1). The highest average recombination rate (7.04 cM/Mb) was in the female map associated with LG14. The lowest average recombination rate (1.10 cM/Mb) was in the male map associated with LG11.

**Fig. 5:**
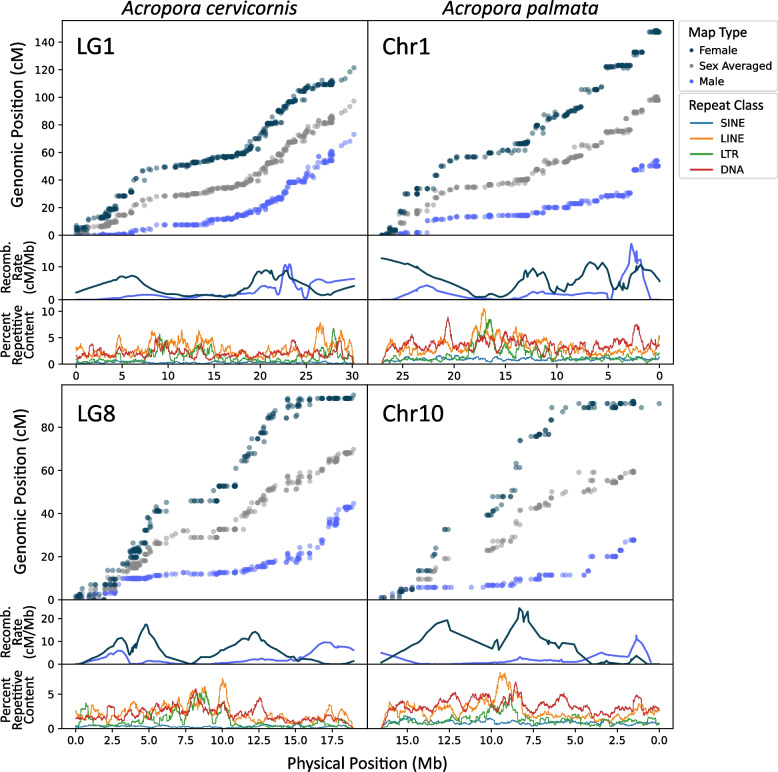
Genetic maps for two homologous pairs of chromosomes of *Acropora cervicornis* and *A. palmata*. LG1 and Chr1 are homologous, as well as LG8 and Chr10. Percent SINE, LINE, LTR, and DNA repeats show putative centromere positions. Repeat content was calculated in 500 Kb sliding windows with 5 Kb steps. Note: *A. palmata* Chr1 and Chr10 x-axes indicating physical position are inverted due to the assembled sequence being reverse of the homologous chromosome in *A. cervicornis*

Recombination landscapes were largely concordant between species, with similar recombination rates and centromere positions, as highlighted in Fig. 5 and Supplementary Fig. 8. However, one homologous chromosome pair (LG11/Chr11) exhibited large differences in map length in which the linkage map for *A. palmata* was almost twice as long as the map for *A. cervicornis*, despite similar physical size (115 cM vs. 61.6 cM in the female map). Female recombination rates were roughly two times as high as male rates in *A. cervicornis* and roughly 2.5 times as high in *A. palmata*.

## Discussion

### Genome comparisons reveal chromosomal macrosynteny despite evolutionary divergence

Here, we present chromosome-scale genome assemblies of the Atlantic acroporids. Our assemblies are similar in quality, genome completeness and repetitive content to other published coral genomes but surpass many in genome contiguity. For example, the comparison of our *A. cervicornis* assembly with the recent genome assembly of another *A. cervicornis* genet from the Florida Keys [123] represents a sevenfold gain in contiguity (as measured by scaffold N_50_); otherwise the two genomes are remarkably similar in gene completeness and assembly size. Both species also possess the most common number of chromosomes amongst acroporids (2*n* = 28 in 72% of species surveyed; [35]), a value concordant with karyotyping in *A. palmata* [156]. The assembly of *A. palmata* is less complete than *A. cervicornis*, as assessed by BUSCO Metazoa v10 [143, 157]. The 87.8% completeness (Supplementary Table 5), compared with 93.1% in *A. cervicornis*, may be the result of small local mis-assemblies introduced during the Hi-C scaffolding process, incomplete polishing, or excessive purging of short, unscaffolded contigs. Nevertheless, the completeness of *A. palmata* is similar to other assemblies derived from similar data types and methods (i.e., short-read primary assembly with additional scaffolding [23, 36]).

Despite their smaller genome sizes, we found that the TE contents of *A. palmata* (16.69%) and *A. cervicornis* (18.01%, Supplementary Table 10), were similar to other acroporid species that had similar contig N50s and were analyzed using the same TE identification methods (namely RepeatModeler and RepeatMasker), ranging from 13.57% in *A. digitifera* to 19.62% in *A. tenuis* (Fig. 4A). These numbers are lower than previous estimates of 40% to 45% for acroporids using a different TE identification method on more fragmented assemblies [23]. Indeed, estimates from dnaPipeTE [109] (an assembly-free approach to TE content estimation) were comparatively much higher than those of RepeatModeler/RepeatMasker (Supplementary Fig. 7, Supplementary Table 10).

Between the Atlantic sister species, genomic synteny is largely conserved despite the relatively deep divergence time of approximately 5 million years [159, 160]. In a broader phylogenetic context, we found agreement with Ying et al. [161] and Shinzato et al. [23], wherein colinear relationships declined with phylogenetic distance within *Acropora* and in comparison with non-acroporids (Fig. 2D, Supplementary Fig. 2 and 3). For example, comparison of the acroporids, members of the complex clade of corals, with the coral *Cataphyllia jardinei*, which belongs to the robust coral clade, show macrosyntenic continuity within the 14 chromosomes (Supplementary Fig. 2A) but gene colinearity was mostly lost (Fig. 2D).

Structural variants were detected between *A. palmata* and *A. cervicornis*, but their presence should be independently confirmed due to the low marker density of the *A. cervicornis* linkage map. This linkage map was used for genome scaffolding and consisted of only 16 markers per Mb and contigs containing a single marker cannot be oriented correctly. Lep-Anchor [105] additionally utilizes long-read data to assist in contig orientation where linkage markers are sparse or absent, but in cases where long reads are too short to span repetitive regions, the correct orientation may still not be resolved. Long distance translocations and large-scale inversions may be more immune to these issues. Additionally, because of the presence of unbridged gaps from Hi-C and linkage scaffolding, break-ends may not be detected or supported by SV callers, despite being detected by alignment dot plots.

The two species discussed here naturally hybridize bidirectionally to form F1 hybrids [47], previously referred to as *A. prolifera*, and rare backcrosses of the F1 with both parent species have been documented. However, F2 generations have not been observed in genetic data from wild colonies [47, 50]. Given the paucity of later generation hybrids (backcrosses and F2s), the hybrids may undergo hybrid breakdown resulting in non-viable or less fit offspring. It is therefore assumed that some post-zygotic genetic mechanism, like differing genomic architectures or speciation genes, exists that represses reproduction between the parental species [50, 162]. For example, large structural variants can cause misalignment during F1 meiosis or death in F2 offspring due to the loss of gene copies required for survival [163]. Such structural variants (SVs) cause F2 sterility in interspecies hybrids of *Drosophila* [164], as well as F2 lethality in wild strains of *Arabidopsis* [165]. Although whole genome alignments between *A. palmata* and *A. cervicornis* demonstrate high levels of macrosynteny and conserved gene colinearity, some regions do exhibit large scale rearrangements (e.g., 2.5 Mb inversion on LG6/Chr4, Fig. 2B and C, Supplementary Table 7). SVs may be acting as a barrier to backcross and F2 offspring formation in the F1 hybrid adults and represent candidates for future studies of hybrid breakdown in this system. However, due to the linkage-based scaffolding used in the *A. cervicornis* assembly, such SVs should be validated using ultra long read or Hi-C sequencing as they may also represent assembly artifacts. Subsequently, population-level sequencing of *A. palmata* and *A. cervicornis* using long-read sequencing technologies could then be employed to assess if SVs represent fixed differences between species that could be linked to hybrid sterility.

In addition to putative SVs, speciation genes may also maintain the reproductive isolation between sister species [162]. Such genes may have a disproportionate effect in driving speciation and are represented by genes such as PRDM9 in mammals [166, 167] and NUP96 in *Drosophila melanogaster* and *D. simulans* [168]. Both PRDM9 and NUP96 cause sterility in F1 hybrids [166, 168]. In our analyses, we have identified genes with nucleotide divergence that significantly exceeds the genomic background (Supplementary Table 8). Such genes were enriched for the GO terms spindle pole (GO:0000922) and midbody (GO:0030406). The meiotic spindle is associated with meiotic drive leading to hybrid male sterility [169] and the formation and remnants of the meiotic midbody are important in developmental competency of mouse oocytes [170]. Such functions may, in part, drive the reproductive barriers isolating *A. palmata* and *A. cervicornis*.

Homologs of suREJ (sperm receptor for egg jelly) and SPAG1 (sperm-associated antigen 1) were also found amongst the most diverged genes between *A. palmata* and *A. cervicornis* (Supplementary Table 8)*.* In sea urchins, suREJ is under positive selection and may be a mechanism to reduce gene flow between conspecifics [171]. Such a gene may play a role in reducing gene flow between *A. palmata* and *A. cervicornis* (as shown in choice/no choice hybridization crosses, [172]). However, suREJ has not been previously associated with hybrid breakdown or hybrid infertility in contrast to SPAG1 [173]. SPAG1 also supports the proper development of oocytes in mouse meiosis [174]. Given these functions in other organisms, SPAG1 may play a role in the bidirectional hybrid breakdown in the Atlantic *Acropora* species. Reciprocal crosses between *A. palmata* and *A. cervicornis* and F2 and backcrosses with the F1 hybrids may provide further insight into the role of SPAG1 in the reproductive isolation of the species.

### Ancestral linkage groups and gene family expansions

Here, we found that over their 52—119 million years (Mya) of history [23], acroporids have retained conserved syntenic gene order to a high degree. While only a small sample size is available for comparison, the maintenance of chromosomal arrangements across deeply diverged coral lineages that split in the Devonian–Carboniferous, approximately 332–357 Mya [175], is surprising. Macrosyntenic patterns gradually degraded and chromosome numbers varied as we compared acroporids to more divergent species from Scleractinia, Actiniaria, Octocorallia, and Medusozoa (Supplementary Fig. 3).

Of the 21 cnidarian- specific ALG arrangements, six were largely intact within the scleractinians (acroporids and *Catalaphyllia*) and one ALG was lost from all but one cnidarian species surveyed. There were seven cases of ALG fusions and one example of centric insertion. *A. millepora* is the only acroporid species where a portion of ALG *G* fused with *L*. This fusion event in *A. millepora* presents an interesting target for further studies in light of the variable hybridization potential among Pacific species within the genus.

Comparison of gene families using presence/absence as well as phylogenetically informed expansions/contraction analyses of identified unique orthogroups at the family level and between the Pacific and Atlantic acroporids. Similar to a prior study [23], the acroporid-specific groups included overrepresentation of gene families involved in coral calcification (galaxin, matrix shell protein and skeletal organic matrix protein) and host-microbe interactions (prosaposin and toll-like receptor) relative to the other cnidarians. A minority of the orthogroups unique to the Atlantic species were annotated and those that were included transposable elements, suggesting numerous coding genes and/or repetitive element copies arose after gene flow stopped between the Atlantic and Pacific acroporids, approximately 2.8 Mya [176, 177]. Notable genes with lineage-specific duplications in the Atlantic acroporids include a gene involved with sperm function (OG0022455: cation channel sperm-associated protein 3), two genes involved in DNA replication (OG0022558: Serine/threonine-protein kinase Nek2 and OG0022391: replication protein A 70 kDa DNA-binding subunit C) and one gene in development (OG0022485: paired box protein). These duplicates should be assessed for functional differences in future studies exploring incompatibility between *A. palmata* and *A. cervicornis*.

In phylogenetically informed analyses, there was an enrichment of ontology terms relating to symbiont-containing vacuoles in the significantly expanding gene families in the Acroporidae. Symbiont-containing vacuole membranes are of crucial function to photosynthetic stony corals (e.g., [178]), of which the family Acroporidae is the most speciose and abundant [179]). It is possible that symbiont-associated gene families were crucial to the evolution and diversification of the family Acroporidae.

Within the genus *Acropora*, gene families that expanded were primarily related to cell–cell adhesion, cell recognition, and nerve components and processes. *Acropora* are unique in their highly complex and intricate morphologies and are the only coral genera to possess distinct axial and radial polyps [180–182]. The evolution of complex colony morphologies associated with the genus *Acropora* may have required neuron-related gene family expansions to help maintain cell-to-cell communication across increasingly complex skeleton morphologies. Retrotransposition terms were also found to be enriched in expanded gene families. Repeat elements represent a significant portion of *Acropora* genomes and these elements may still be dynamic in nature, despite not substantially altering genome size. In the absence of significant repeat expansions, further work is required to determine if retrotransposition drives copy number variants within populations of *Acropora* given the significant enrichment of terms associated with retrotransposition and viral response. Heat stress-dependent retrotransposition is prevalent in the coral symbiont *Symbiodinium microadriaticum*, and similar processes may be at play within the host species [183].

### Heterochiasmy and recombination landscapes of the Caribbean acroporids

Heterochiasmy in *A. palmata* and *A. cervicornis* was among the most pronounced estimates observed in plants or animals [184]. Generally, recombination rates were higher in *A. palmata*, potentially due to differences in overall assembly length. The k-mer estimated genome sizes were similar (333 Mb in *A. palmata* and 331 Mb in *A. cervicornis*, Supplementary Table 4) but assembly sizes were more variable, with *A. palmata* being 287 Mb and *A. cervicornis* being 305 Mb. This would result in increased genome-wide *A. palmata* recombination rates simply due to assembly size. However, regardless of assembly sizes, genetic map lengths are greater in *A. palmata* (consensus map length 1013 cM) than in *A. cervicornis* (927 cM). Based on repeat density and local recombination rates (i.e. regions with elevated repeat content and suppressed recombination, as described in Hartley and O’Neill [185] and Schreiber et al. [186]), all chromosomes in both species appear to be metacentric or submetacentric (Fig. 5 and Supplementary Fig. 8), like in the Pacific acroporid, *Acropora pruinosa* [187]. Centromeric regions appear to be associated with long interspersed nuclear element (LINE) repeats, as shown by the prominent peaks in LINE content.

Within chromosomes, both species exhibit commonly observed local recombination landscapes (e.g., higher local recombination rates in females across whole chromosomes or higher recombination in males near telomeres; [58]). Twelve out of fourteen chromosomes exhibit recombination landscapes where local female rates are generally higher than male rates throughout the chromosome. Female maps exhibit marked declines in recombination around the presumed centromere while males show low, chromosome-wide recombination. However, in two cases, male local recombination rates are higher than female rates at one end of the chromosome, in telomeric regions (LG9/Chr9, LG8/Chr10, Fig. 5 and Supplementary Fig. 8).

Comparing recombination among the three acroporid species revealed numerous similarities among the Atlantic (*A. palmata, A. cervicornis*) and Pacific (*A. millepora*) corals. In all three acroporid linkage maps, the overall female map length was longer than the male length. However, the higher recombination in the female map in *A. millepora* was driven by only a subset of linkage groups [60]. In *A. palmata* and *A. cervicornis*, we find that the pattern is consistent across all chromosomes (Table 1, Fig. 5, and Supplementary Fig. 8).

The underlying causes of heterochiasmy are an active area of exploration in genetics [58, 188, 189]. Heterochiasmy was thought to be driven by the presence of sex chromosomes, but this is contradicted by similar patterns of heterochiasmy in simultaneous hermaphrodites that lack sex chromosomes (such as *A. palmata* and *A. cervicornis*), as well as species with environmental sex determination [57, 62, 190]. Other proposed explanations for heterochiasmy include genetic drift [191], haploid selection [188, 192], and female meiotic drive [184, 193]. Due to some consistency in patterns across divergent taxonomic groups, drift has been regarded as an unlikely explanation [58]. Wang et al. [60] concluded that haploid selection was the most likely culprit in the coral *A. millepora* at a time when theory for the role of meiotic drive had not yet been developed. There has been conflicting evidence for the role of haploid selection in animals, which have fewer expressed genes in sperm and eggs compared to plants [189, 194]. Higher recombination rates adjacent to centromeres in female maps suggest that meiotic drive may also be a potential mechanism for heterochiasmy in the Atlantic *Acropora*. The presence of drive may either serve to suppress or favor recombination in the female close to the centromeres depending on whether it occurs during Meiosis I or Meiosis II [184, 193]. During Meiosis I, selective pressure for increased recombination around the centromere is expected to reduce the spread of harmful drive alleles by decoupling them from the centromere [184].

In bivalves, sessile organisms with similar reproductive strategies to corals, the global patterns of recombination are similar to the Atlantic *Acropora* with females exhibiting higher rates than those of males, with locally higher recombination rates at centromeres in females and locally higher recombination rates at telomeres in males [58]. We hypothesize that there may be bioenergetic constraints on the production of each gamete which limit the frequency of recombination in sperm, particularly in broadcast spawning marine organisms (such as corals and bivalves) that may produce billions of sperm but only few eggs in each reproductive event. In *Acropora*, a colony may produce thousands of gamete bundles containing eggs and sperm. Each bundle contains 4–6 eggs and ~ 100,000 to ~ 150,000 sperm cells [195]. This is in comparison with insects, which have higher male recombination rates when compared with females, and produce as few as 50 sperm cells per reproductive event and may produce fewer sperm in their entire lifetime than a single coral gamete bundle [196–199]. Additional hypotheses proposed by Sardell et al. also involve the differing life history traits (e.g., sex organ temperature or aging) of each sex. As the species discussed here are hermaphroditic, we do not think these hypotheses are likely to explain the heterochiasmy of the Atlantic acroporids.

The average genome-wide recombination rates for *A. palmata* and *A. cervicornis* (3.04 to 3.5 cM/Mb) are higher than the average recombination rates for animals (2.54 cM/Mb) [57]. Average recombination rates for the two species are similar to the rates for insects, crustaceans, and fish, but higher than the averages for groups such as birds, amphibians, reptiles, and mammals [57]. This may indicate a rapid response to selection in acroporids because the proportion of substitutions fixed by adaptive evolution is positively correlated with recombination rate [200]. Future work comparing recombination rates across coral populations and taxa would be valuable in clarifying the evolutionary consequences of these patterns.

The lifespan of corals may also play a role in their overall high recombination rate compared with other metazoans. Corals are amongst the longest living metazoans, with some species capable of living thousands of years (e.g., *Acropora palmata* [156]). Given that reef-building corals are sessile and can live for thousands of years but typically only reproduce once per year in mass spawning events (e.g., [201]), high recombination rates may maximize the frequency at which favorable gene combinations are produced [202, 203] to match changing environments.

The local and genome-wide recombination rates calculated from the genetic linkage maps for *A. palmata* and *A. cervicornis* provide novel insights into the recombination landscape of corals. The density of markers in this resource now opens the possibility for quantitative trait locus (QTL) analyses as well as more precise haplotype imputation and genetic association studies in these species (Fig. 5). QTL mapping allows for the identification of loci that have consistent, predictable effects on phenotype across individuals. In plants, this is frequently used to assist with breeding programs [204]. As populations of many corals have rapidly declined [205], such a tool could assist in the design of restoration approaches. Additionally, phasing and imputation software commonly used in genome-wide association studies (GWAS) such as BEAGLE [206], GLIMPSE2 [207], and SHAPEIT [208] take into account recombination rates across chromosomes to more accurately statistically phase and impute data. The generation of these assemblies and genetic maps now enables complex genetic association studies not previously possible in these threatened non-model organisms. With these data, we have also demonstrated the application of the *Acropora* SNP array [101] as a successful genotyping method for the generation of a genetic linkage map, which provides a cost-effective means for creating additional maps for the F1 hybrids of *A. palmata* and *A. cervicornis*.

## Conclusions

The genomic resources presented here revealed that the adaptive capacity of endangered Atlantic *Acropora* corals is likely not hindered by their recombination rates, as both species exhibit high genome-wide recombination rates with prominent heterochiasmy between sexes in these simultaneous hermaphrodites. Moreover, the sister species exhibit remarkable levels of macrosynteny and gene colinearity with one another as well as with the Pacific species, especially considering the > 50 Mya history of the genus. Our assemblies suggest that, like many scleractinians, the Atlantic acroporid genomes consist of 14 chromosomes; a derived state compared to the last common ancestor of the Cnidaria which is proposed to have had 17 chromosomes [4]. The conserved number of haploid chromosomes among many, but not all, of the acroporids is 14 (2*n* = 28, [35]) and the high level of macrosynteny across the *Acropora* genus may enable these syngameons described above. In the Pacific, it has been suggested that hybridization acts as an evolutionary force driving speciation [209, 210]. However, in the Atlantic *Acropora*, hybridization between the two sister species yields an F1 hybrid with limited fertility [50] despite the high levels of macrosynteny and gene colinearity of their genomes. In this study, we highlight putative genes and gene families that may drive reproductive isolation of the two Atlantic sister species. Further experimental work is required to gather further support for these targets. Together, the chromosome-scale assemblies and genetic maps we present here are the first detailed look at the genomic landscapes of these critically endangered species. The availability of these genomic resources helps facilitate genome-wide association studies and discovery of quantitative trait loci which can aid in the conservation of endangered corals.

## Supplementary Information

Additional file 1: Supplementary Table 1: Summary of sequencing libraries used for genome assembly of the Atlantic acroporids. All data and assemblies are available at NCBI BioProject PRJNA473816.

Additional file 2: Supplementary Table 2: Genomic resources used to compare gene content and syntenic arrangement. Genome assemblies and their predicted proteins were downloaded from sources noted below. Taxonomy is ranked as Subphylum, Class and Order.

Additional file 3: Supplementary Table 3: Assembly statistics for the genome assemblies of the acroporids compared in this study.

Additional file 4: Supplementary Table 4: K-mer genome size estimates. Three different k-mer based genome size prediction tools were used: GenomeScope2, findGSE and kmergenie. The first two tools used the k-mer distribution histogram generated by Jellyfish with a k-mer size of 119. bp = base pairs.

Additional file 5: Supplementary Table 5: Assembly statistics showing completeness and contiguity of Acropora cervicornis HG0582 [123] and HG0005 assemblies (this study). Mb = megabases. BUSCO = Benchmarking Universal Single-Copy Orthologs.

Additional file 6: Supplementary Table 6: Comparison of Acropora palmata Hi-C (pseudo) chromosomes that are homologous to A. cervicornis linkage groups. Mb = megabases.

Additional file 7: Supplementary Table 7: Number of structural variants identified between Acropora palmata and A. cervicornis genome assemblies. N/A = not applicable.

Additional file 8: Supplementary Table 8: Outlier genes between Acropora palmata and A. cervicornis in terms of sequence identity. Percent identity was calculated using rustybam [140] in windows corresponding with gene coordinates using whole genome PAF alignments derived from minimap2 [92]. Genes were considered outliers if their percent identity was below Q1 – (3*IQR).

Additional file 9: Supplementary Table 9: Tracing the 21 cnidarian ancestral linkage groups as defined by Simakov et al. 2022 in acroporid corals. x = chromosomal fusion. Acroporids share the same ALG architecture except for ALG L which is fused to G in A. millepora only. AHYA = Acropora hyacinthus , APAL = A. palmata , AMIL = A. millepora , ACER = A. cervicornis , CJAR = Catalaphyllia jardinei , NVEC = Nematostella vectensis , SSPP = Scolanthus sp. , XSPP = Xenia sp. , HVUL = Hydra vulgaris , HSYM = Hydractinia symbiolongicarpus , CXAM = Cassiopea xamachana.

Additional file 10: Supplementary Table 10: Summary of repeat content in Acropora palmata and A. cervicornis genome assemblies. Bolded transposable elements are abundant among all acroporids.

Additional file 11: Supplementary Fig. 1: Alignments of the two available Acropora cervicornis genome assemblies. Genet K2 assembly (STAGdb ID HG0582, Selwyn and Vollmer [123] is compared with the genet M5 assembly (STAGdb ID HG0005, this study). A) Assembly-assembly minimap2 alignments > 1Mbp and their chromosome locations, plotted with pafr. B) Dot plots comparing the HG0582 assembly with the 14 HG0005 chromosomes (plotted with dotplotly, [135]). Contigs are largely concordant with the exception of one large HG0582 contig that was split across HG0005 chromosomes.

Additional file 12: Supplementary Fig. 2: Oxford dot plots of scleractinian chromosomes and 29 ancestral linkage groups. Scleractinian chromosomes are plotted against A. cervicornis chromosomes (A) and of acroporids chromosomes against 29 ancestral linkage groups (ALGs) of last common ancestor of sponges, cnidarians and bilaterians [2] (B).

Additional file 13: Supplementary Fig. 3: Oxford dot plot of cnidarian assemblies against A. cervicornis chromosomes, arranged by taxonomic groups. Ancestral linkage groups proposed for the last common ancestor of Cnidaria, Bilateria and sponges [2] are indicated by color.

Additional file 14: Supplementary Fig. 4: Significantly enriched GO terms in expanding or contracting gene families of Acroporidae. Top 10 cell component, biological process, and molecular function GO terms associated with phylogenetically significant expand (orange) and contracting (blue) gene families for the family Acroporidae.

Additional file 15: Supplementary Fig. 5: Significantly enriched GO terms in expanding or contracting gene families of Acropora . Top 10 cell component, biological process, and molecular function GO terms associated with phylogenetically significant expand (orange) and contracting (blue) gene families for the genus Acropora.

Additional file 16: Supplementary Fig. 6: Significantly enriched GO terms in expanding or contracting gene families of the Atlantic Acropora . Top 10 cell component, biological process, and molecular function GO terms associated with phylogenetically significant expand (orange) and contracting (blue) gene families for the Atlantic Acropora species.

Additional file 17: Supplementary Fig. 7: Total repeat content estimates based on short-read sequence data of the Atlantic acroporids. Repeat classes are colored as red = DNA, green = LTR, yellow = LINE, blue = SINE, purple = Helitron, and gray = Other (i.e., satellites, simple repeats, unannotated TEs).

Additional file 18: Supplementary Fig. 8: Full linkage and recombination maps for all homologous chromosomes in the Atlantic acroporids. LINE content appears to peak in regions with low recombination rates, highlighting centromere positions.

## Data Availability

Genome assemblies, gene predictions, functional annotations, and genetic maps are publicly available at https://zenodo.org/doi/10.5281/zenodo.12021086. NCBI genome accessions are GCA_025960835.2 for *A. palmata*, GCA_037043185.1 for *A. cervicornis* version 1, and GCA_041430625.1 for *A. cervicornis* version 2. All raw data and assemblies have been deposited in NCBI BioProject PRJNA473816 and all SRA accessions are provided in Supplementary Table 1.
