# Supplementary figures and images for: Chromosome-level genome assemblies and genetic maps reveal heterochiasmy and macrosynteny in endangered Atlantic Acropora
Source: BMC Genomics. 2024 Nov 20;25:1119. doi: 10.1186/s12864-024-11025-3 (PMC11577847; doi:10.1186/s12864-024-11025-3)

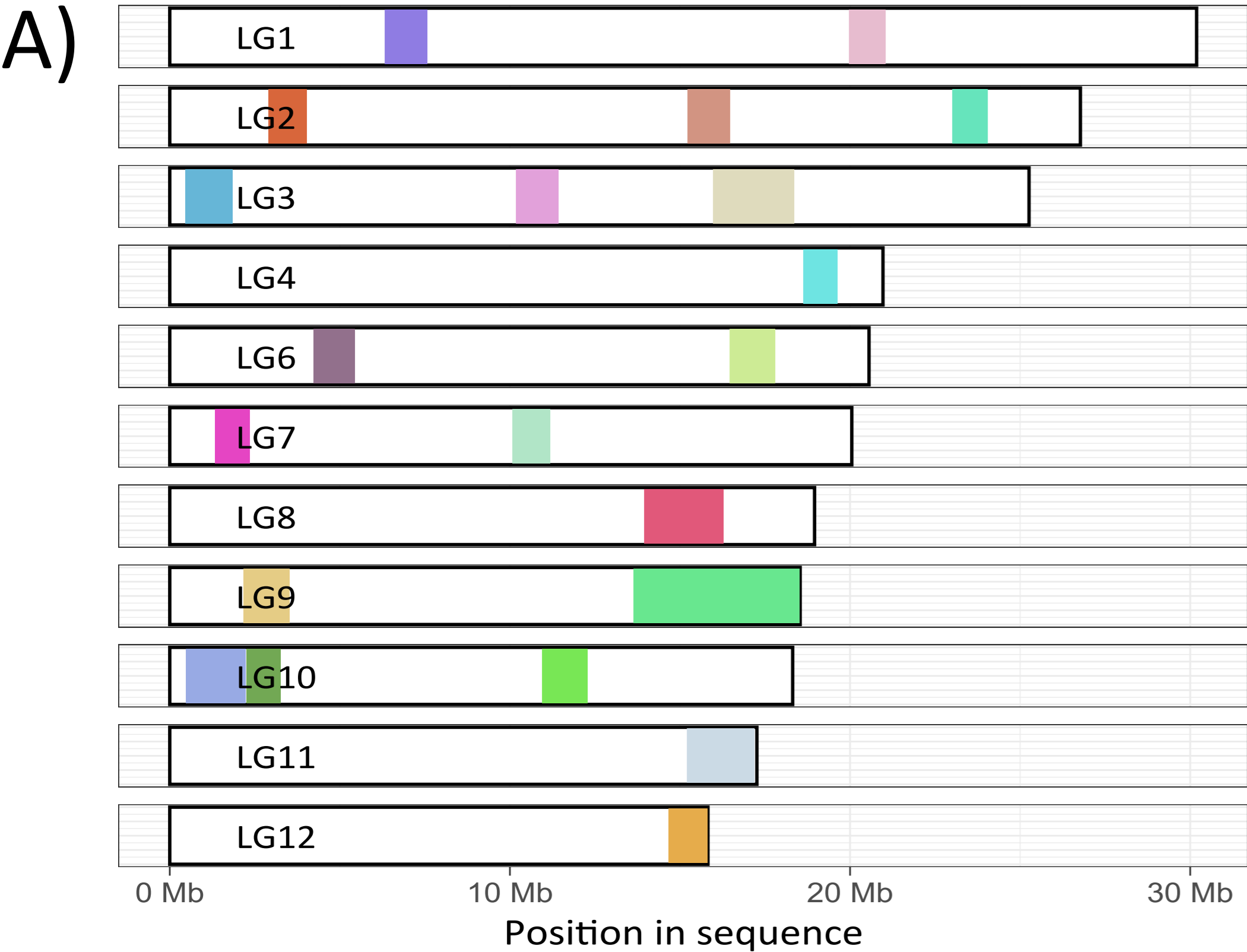

Query Name (Selwyn and Vollmer 2023)

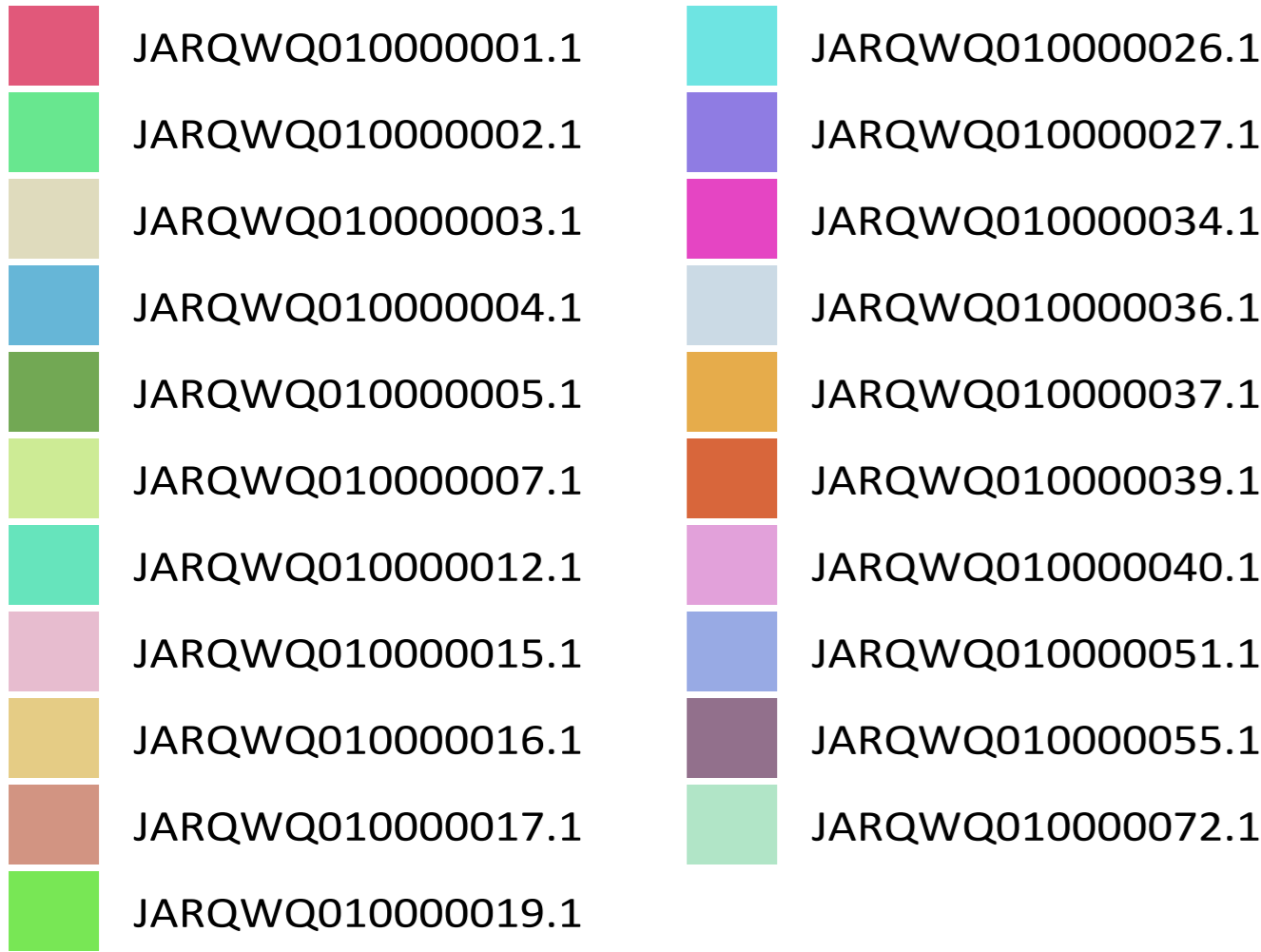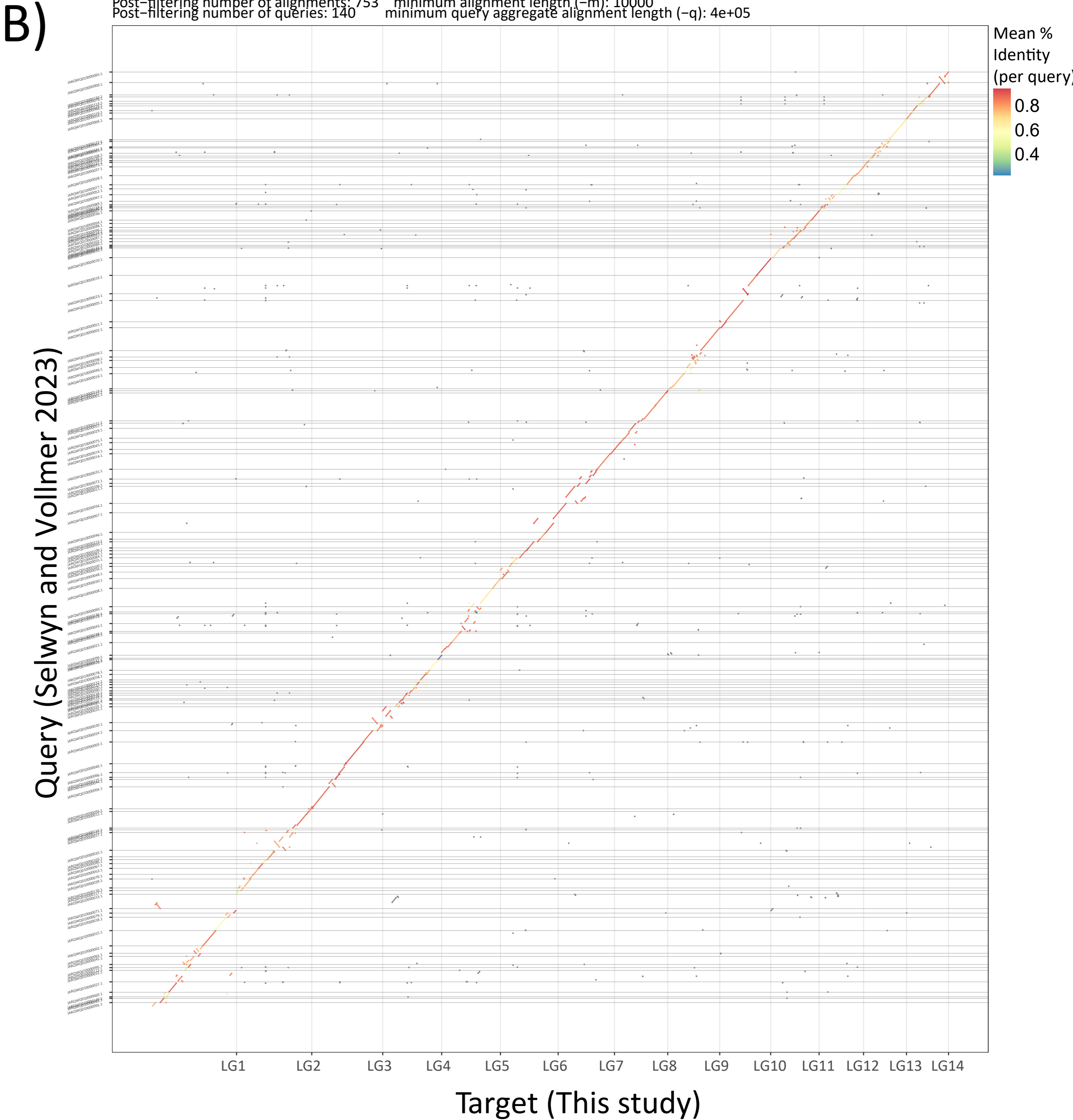

Supplement: Supplementary file 11 — Additional file 11: Supplementary Fig. 1: Alignments of the two available Acropora cervicornis genome assemblies. Genet K2 assembly (STAGdb ID HG0582, Selwyn and Vollmer [123] is compared with the genet M5 assembly (STAGdb ID HG0005, this study). A) Assembly-assembly minimap2 alignments > 1Mbp and their chromosome locations, plotted with pafr. B) Dot plots comparing the HG0582 assembly with the 14 HG0005 chromosomes (plotted with dotplotly, [135]). Contigs are largely concordant with the exception of one large HG0582 contig that was split across HG0005 chromosomes. [file 12864_2024_11025_MOESM11_ESM.pdf]

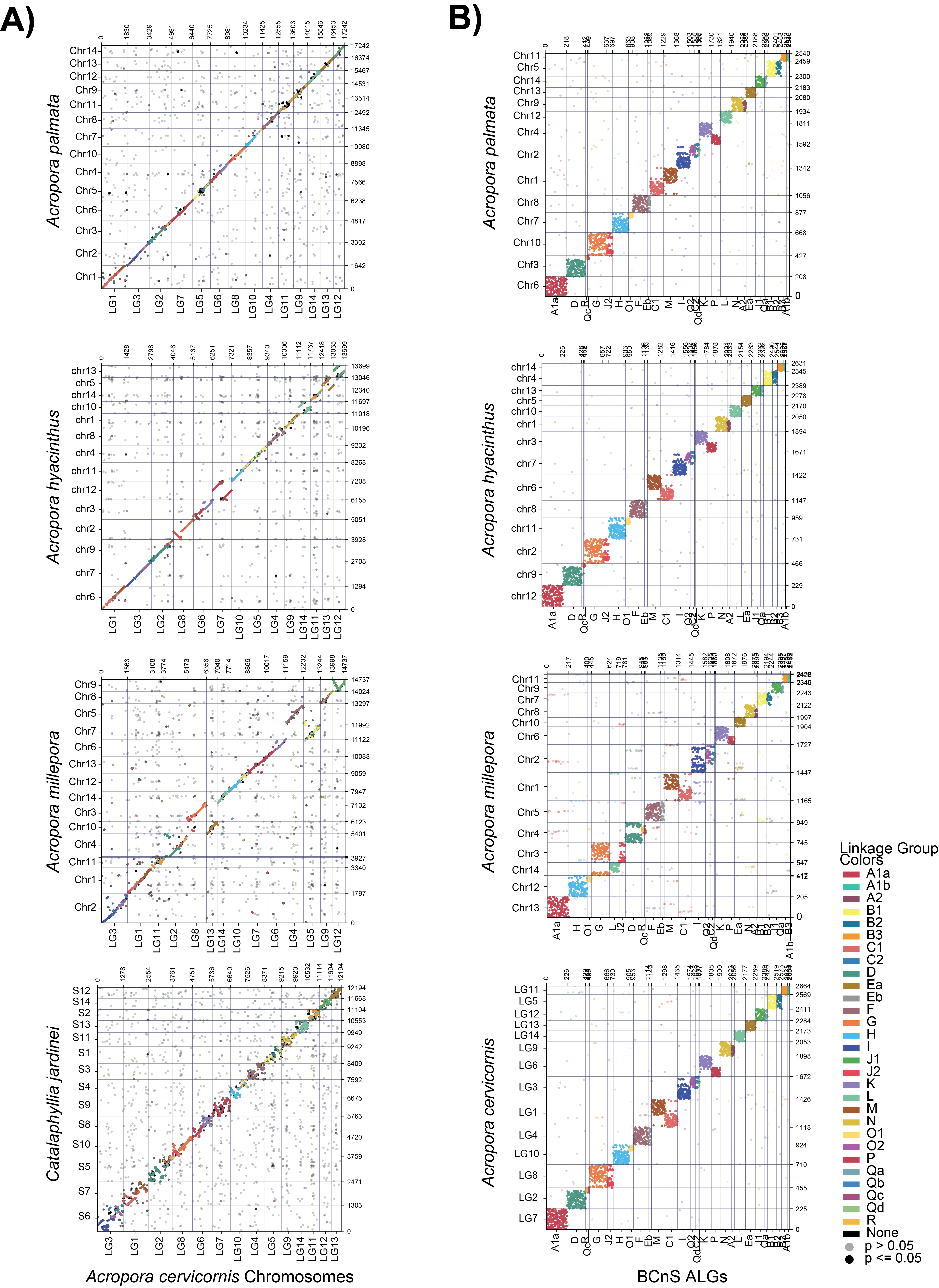

Supplement: Supplementary file 12 — Additional file 12: Supplementary Fig. 2: Oxford dot plots of scleractinian chromosomes and 29 ancestral linkage groups. Scleractinian chromosomes are plotted against A. cervicornis chromosomes (A) and of acroporids chromosomes against 29 ancestral linkage groups (ALGs) of last common ancestor of sponges, cnidarians and bilaterians [2] (B). [file 12864_2024_11025_MOESM12_ESM.png]

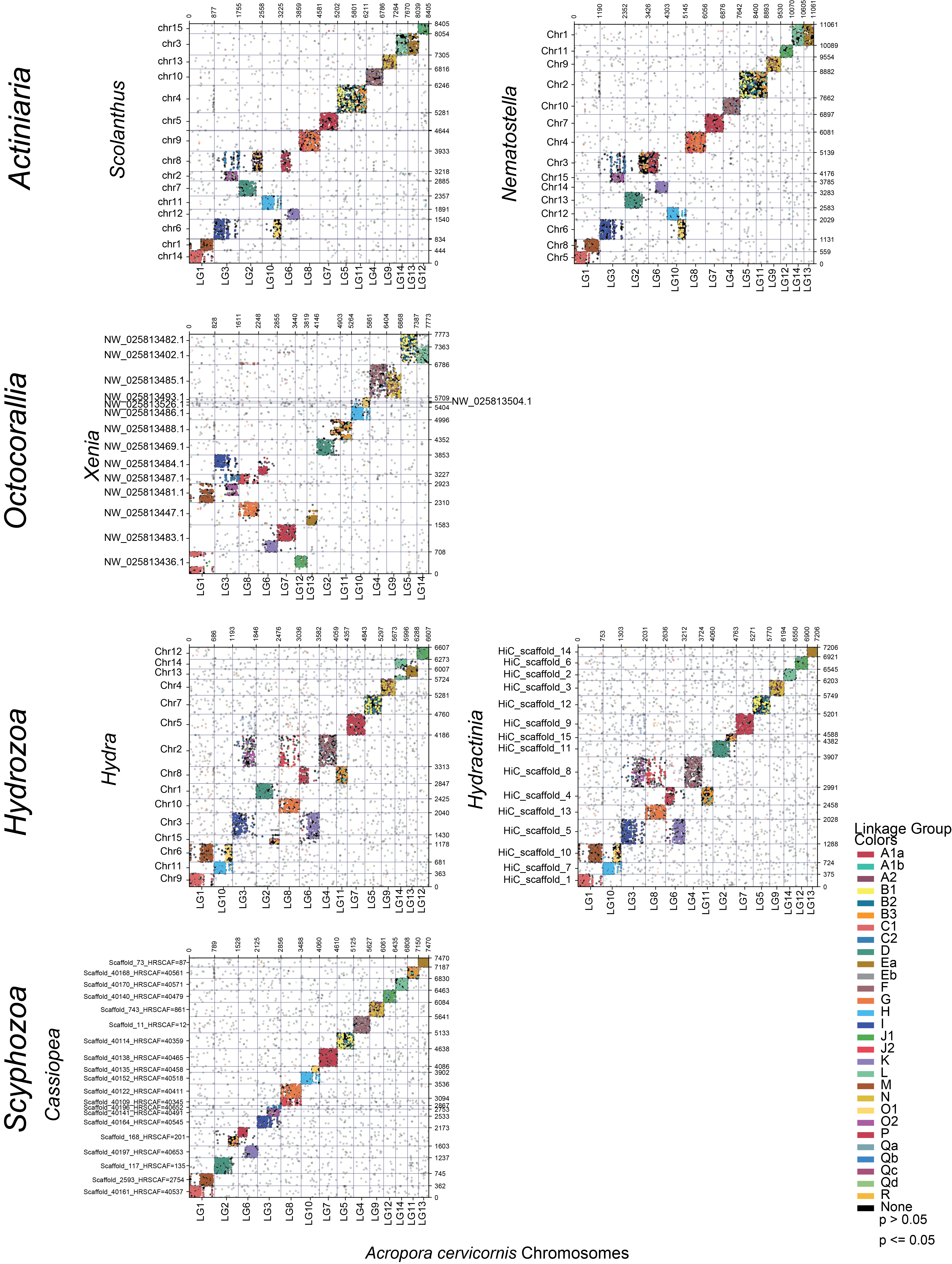

Supplement: Supplementary file 13 — Additional file 13: Supplementary Fig. 3: Oxford dot plot of cnidarian assemblies against A. cervicornis chromosomes, arranged by taxonomic groups. Ancestral linkage groups proposed for the last common ancestor of Cnidaria, Bilateria and sponges [2] are indicated by color. [file 12864_2024_11025_MOESM13_ESM.png]

# Acroporidae

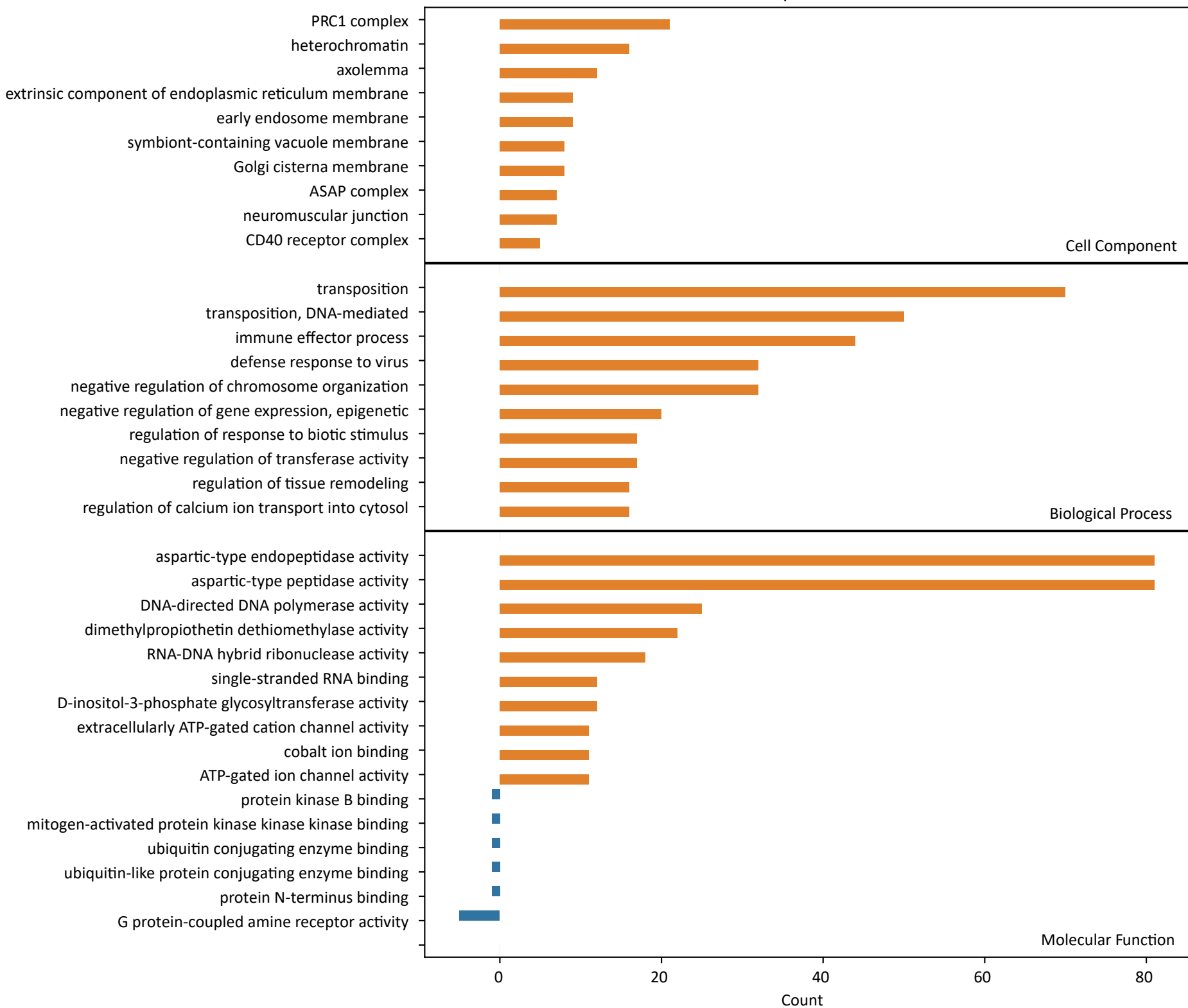

Supplement: Supplementary file 14 — Additional file 14: Supplementary Fig. 4: Significantly enriched GO terms in expanding or contracting gene families of Acroporidae. Top 10 cell component, biological process, and molecular function GO terms associated with phylogenetically significant expand (orange) and contracting (blue) gene families for the family Acroporidae. [file 12864_2024_11025_MOESM14_ESM.pdf]

# Atlantic Acropora

Cell Component

Biological Process

Molecular Function

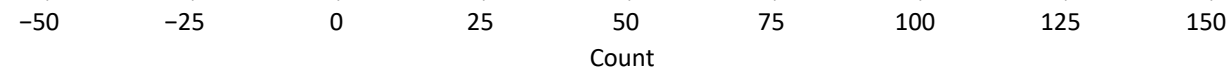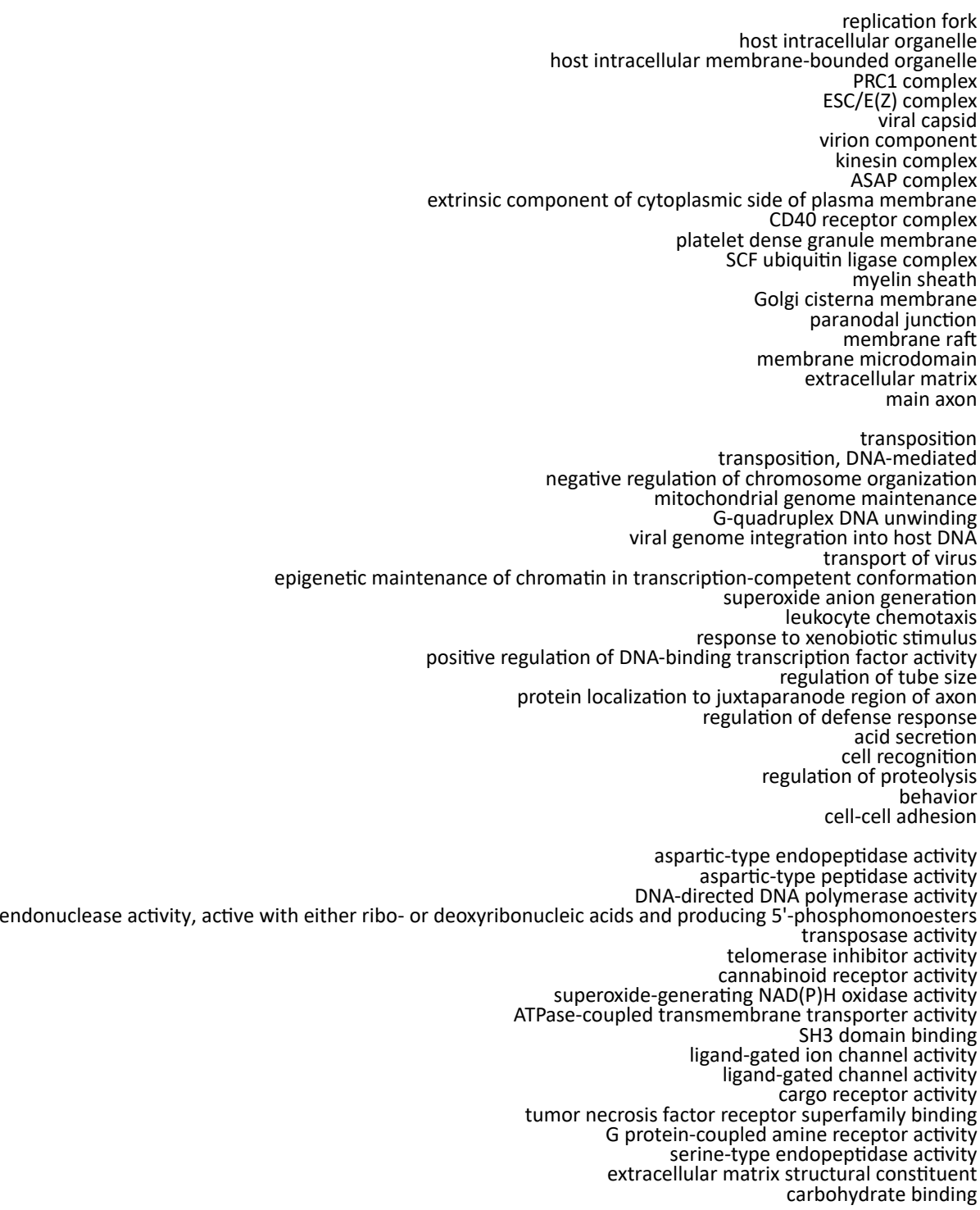

Supplement: Supplementary file 16 — Additional file 16: Supplementary Fig. 6: Significantly enriched GO terms in expanding or contracting gene families of the Atlantic Acropora . Top 10 cell component, biological process, and molecular function GO terms associated with phylogenetically significant expand (orange) and contracting (blue) gene families for the Atlantic Acropora species. [file 12864_2024_11025_MOESM16_ESM.pdf]

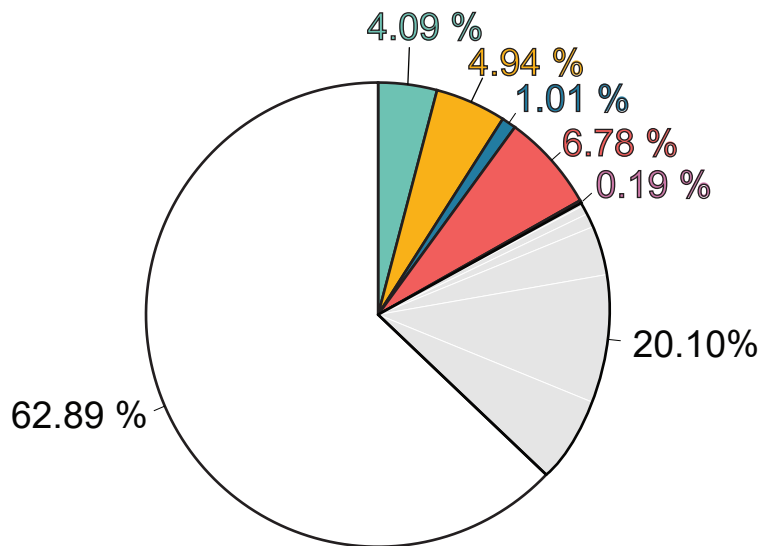

*A. palmata*

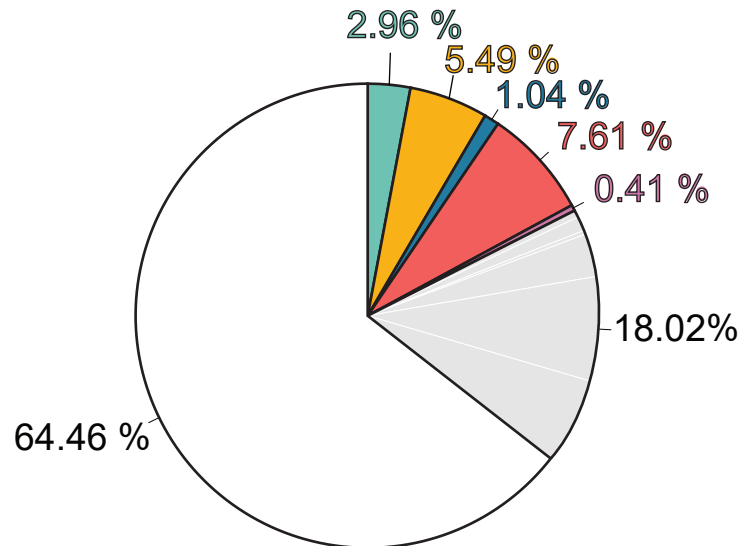

*A. cervicornis*

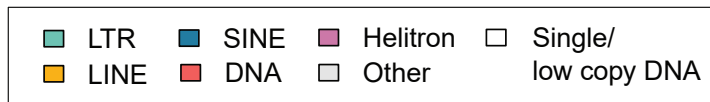

Supplement: Supplementary file 17 — Additional file 17: Supplementary Fig. 7: Total repeat content estimates based on short-read sequence data of the Atlantic acroporids. Repeat classes are colored as red = DNA, green = LTR, yellow = LINE, blue = SINE, purple = Helitron, and gray = Other (i.e., satellites, simple repeats, unannotated TEs). [file 12864_2024_11025_MOESM17_ESM.pdf]
