# Supplementary material for: Chromosome-level genome assemblies and genetic maps reveal heterochiasmy and macrosynteny in endangered Atlantic Acropora
Source: BMC Genomics. 2024 Nov 20;25:1119. doi: 10.1186/s12864-024-11025-3 (PMC11577847; doi:10.1186/s12864-024-11025-3)
Supplement: Supplementary file 15 — Additional file 15: Supplementary Fig. 5: Significantly enriched GO terms in expanding or contracting gene families of Acropora . Top 10 cell component, biological process, and molecular function GO terms associated with phylogenetically significant expand (orange) and contracting (blue) gene families for the genus Acropora. [file 12864_2024_11025_MOESM15_ESM.pdf]

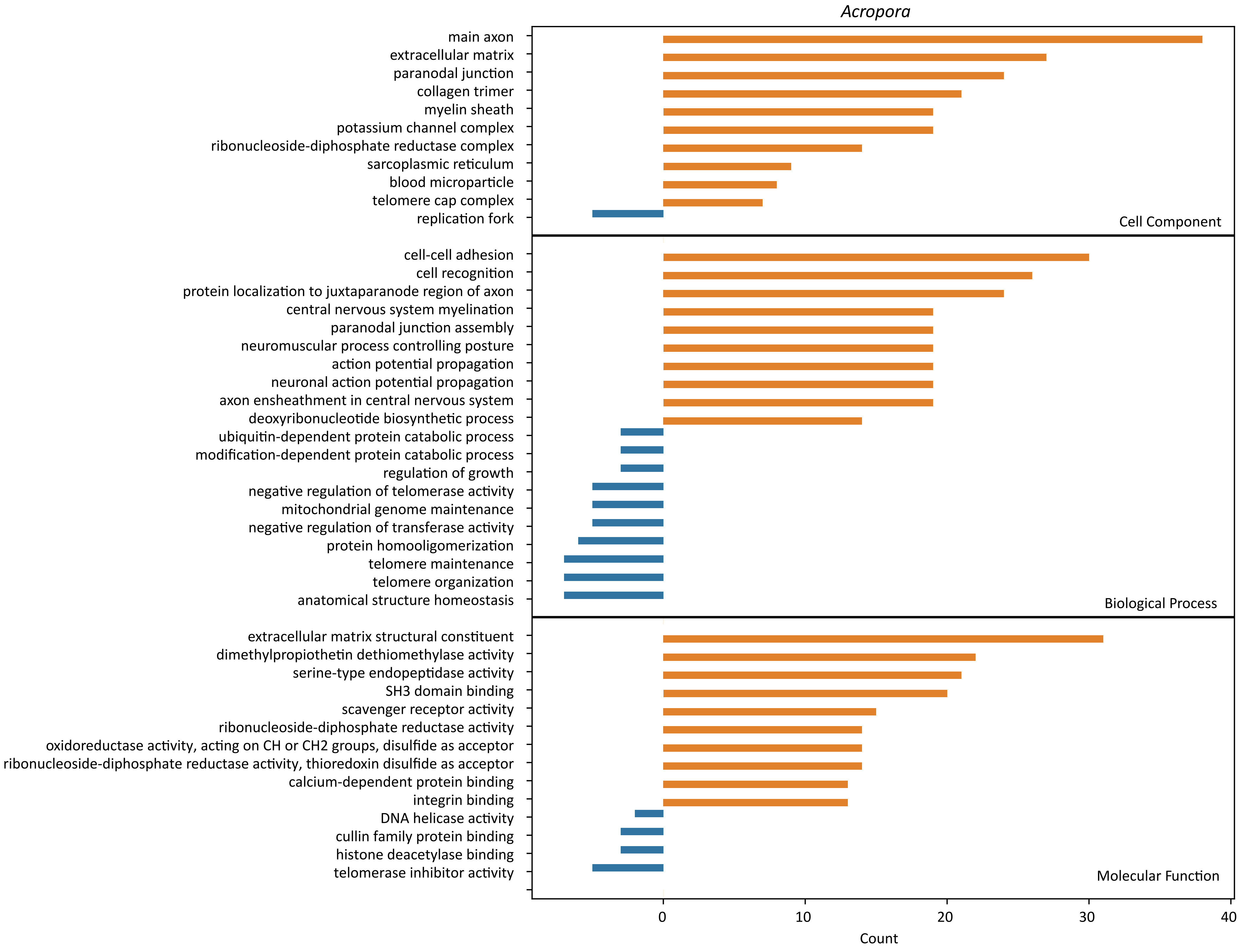

Acropora
main axon
extracellular matrix
paranodal junction
collagen trimer
myelin sheath
potassium channel complex
ribonucleoside-diphosphate reductase complex
sarcoplasmic reticulum
blood microparticle
telomere cap complex
replication fork
Cell Component
cell-cell adhesion
cell recognition
protein localization to juxtaparanode region of axon
central nervous system myelination
paranodal junction assembly
neuromuscular process controlling posture
action potential propagation
neuronal action potential propagation
axon ensheathment in central nervous system
deoxyribonucleotide biosynthetic process
ubiquitin-dependent protein catabolic process
modification-dependent protein catabolic process
regulation of growth
negative regulation of telomerase activity
mitochondrial genome maintenance
negative regulation of transferase activity
protein homooligomerization
telomere maintenance
telomere organization
anatomical structure homeostasis
Biological Process
extracellular matrix structural constituent
dimethylpropiothetin dethiomethylase activity
serine-type endopeptidase activity
SH3 domain binding
scavenger receptor activity
ribonucleoside-diphosphate reductase activity
oxidoreductase activity, acting on CH or CH2 groups, disulfide as acceptor
ribonucleoside-diphosphate reductase activity, thioredoxin disulfide as acceptor
calcium-dependent protein binding
integrin binding
DNA helicase activity
cullin family protein binding
histone deacetylase binding
telomerase inhibitor activity
Molecular Function
0
10
20
30
40
Count
